# LR_Gapcloser: a tiling path-based gap closer that uses long reads to complete genome assembly

**DOI:** 10.1093/gigascience/giy157

**Published:** 2018-12-21

**Authors:** Gui-Cai Xu, Tian-Jun Xu, Rui Zhu, Yan Zhang, Shang-Qi Li, Hong-Wei Wang, Jiong-Tang Li

**Affiliations:** Key Laboratory of Aquatic Genomics, Ministry of Agriculture and Rural Affairs, CAFS Key Laboratory of Aquatic Genomics and Beijing Key Laboratory of Fishery Biotechnology, Chinese Academy of Fishery Sciences, 150 Yongding Road, Beijing, 100141, China; College of Marine Science, Zhejiang Ocean University, 1 Haida South Road, Zhoushan, 316022, China; College of Marine Science, Zhejiang Ocean University, 1 Haida South Road, Zhoushan, 316022, China; Key Laboratory of Aquatic Genomics, Ministry of Agriculture and Rural Affairs, CAFS Key Laboratory of Aquatic Genomics and Beijing Key Laboratory of Fishery Biotechnology, Chinese Academy of Fishery Sciences, 150 Yongding Road, Beijing, 100141, China; College of Fisheries and Life Science, Shanghai Ocean University, 999 Huchenghuan Road, Shanghai, 201306, China; Key Laboratory of Aquatic Genomics, Ministry of Agriculture and Rural Affairs, CAFS Key Laboratory of Aquatic Genomics and Beijing Key Laboratory of Fishery Biotechnology, Chinese Academy of Fishery Sciences, 150 Yongding Road, Beijing, 100141, China; Key Laboratory of Aquatic Genomics, Ministry of Agriculture and Rural Affairs, CAFS Key Laboratory of Aquatic Genomics and Beijing Key Laboratory of Fishery Biotechnology, Chinese Academy of Fishery Sciences, 150 Yongding Road, Beijing, 100141, China; Key Laboratory of Aquatic Genomics, Ministry of Agriculture and Rural Affairs, CAFS Key Laboratory of Aquatic Genomics and Beijing Key Laboratory of Fishery Biotechnology, Chinese Academy of Fishery Sciences, 150 Yongding Road, Beijing, 100141, China; Key Laboratory of Aquatic Genomics, Ministry of Agriculture and Rural Affairs, CAFS Key Laboratory of Aquatic Genomics and Beijing Key Laboratory of Fishery Biotechnology, Chinese Academy of Fishery Sciences, 150 Yongding Road, Beijing, 100141, China

**Keywords:** gap-closure, genome assembly, third-generation sequencing, next-generation sequencing, repetitive elements

## Abstract

**Background:**

Completing a genome is an important goal of genome assembly. However, many assemblies, including reference assemblies, are unfinished and have a number of gaps. Long reads obtained from third-generation sequencing (TGS) platforms can help close these gaps and improve assembly contiguity. However, current gap-closure approaches using long reads require extensive runtime and high memory usage. Thus, a fast and memory-efficient approach using long reads is needed to obtain complete genomes.

**Findings:**

We developed LR_Gapcloser to rapidly and efficiently close the gaps in genome assembly. This tool utilizes long reads generated from TGS sequencing platforms. Tested on *de novo* assembled gaps, repeat-derived gaps, and real gaps, LR_Gapcloser closed a higher number of gaps faster and with a lower error rate and a much lower memory usage than two existing, state-of-the art tools. This tool utilized raw reads to fill more gaps than when using error-corrected reads. It is applicable to gaps in the assemblies by different approaches and from large and complex genomes. After performing gap-closure using this tool, the contig N50 size of the human CHM1 genome was improved from 143 kb to 19 Mb, a 132-fold increase. We also closed the gaps in the *Triticum urartu* genome, a large genome rich in repeats; the contig N50 size was increased by 40%. Further, we evaluated the contiguity and correctness of six hybrid assembly strategies by combining the optimal TGS-based and next-generation sequencing-based assemblers with LR_Gapcloser. A proposed and optimal hybrid strategy generated a new human CHM1 genome assembly with marked contiguity. The contig N50 value was greater than 28 Mb, which is larger than previous non-reference assemblies of the diploid human genome.

**Conclusions:**

LR_Gapcloser is a fast and efficient tool that can be used to close gaps and improve the contiguity of genome assemblies. A proposed hybrid assembly including this tool promises reference-grade assemblies. The software is available at http://www.fishbrowser.org/software/LR_Gapcloser/.

## Introduction

Next-generation sequencing (NGS) technologies allow for the low-cost and high-speed construction of genome sequences by *de novo* assembly. Along with the advantages of NGS technologies, over the last decade, many genome projects (e.g., the 10K Genome Project [1] and 100K Pathogen Genome Project [2]) were initiated and the genomes of numerous species were assembled [3, 4]. However, factors such as sequencing biases [5], repeat regions [6], and heterochromatin [7] make some regions difficult or impossible to assemble, leading to gaps and fragmented genome assemblies.

The gap-closure process is the last but most essential step for increasing the completeness and contiguity of genome assemblies. To obtain complete genomes, several gap-closure approaches, including GapFiller [8], GapCloser [9], Sealer [10], GapBlaster [11], GapReduce [12], and Gap2Seq [13], were developed to use NGS reads or pre-assembled contigs [14] to fill the gaps. However, these gap-closing tools display a high rate of misassembly during the gap-closing process [15]. Furthermore, it is hard to close all gaps, especially large ones, by using these tools. Long single-molecule sequencing technologies, also known as third-generation sequencing (TGS) technologies, for instance, Pacific Biosciences (PacBio) and Nanopore platforms, produce long and unbiased reads [16, 17] that have the potential to fill these gaps and achieve complete genome assemblies. PBJelly [18] and GMcloser [15] employ PacBio reads for gap closure. PBJelly aligns long reads to the reference assemblies using basic local alignment with successive refinement [19], selects supporting reads, performs local gap assembly, and decides upon an accurate assembly for gap filling. GMcloser splits scaffolds into sub-contigs and aligns long reads to sub-contigs using MUMmer [20] or blastn (Basic local alignment search tool). GMcloser uses likelihood-based classifiers to correctly assign long reads to gaps in scaffolds. However, their disadvantages, including long runtime, high memory usage and low closure performance, limit their application, especially to large and complex genomes. Therefore, a fast and memory-efficient gap-closure approach is required to fill gaps in genome assemblies.

Here, we developed LR_Gapcloser to efficiently and rapidly close gaps in assemblies using long reads. Many notable advantages were exhibited compared to previous gap-closing tools; these include higher gap-closure performance, less runtime, fewer peak memories, and fewer misassemblies. Even for both large and complex genomes and repeat-derived gaps, this tool exhibited better performance. Finally, for the genomes that were sequenced using both NGS and TGS technologies, we evaluated the contiguity and correctness of different hybrid assembly strategies and proposed an optimal hybrid strategy combining TGS-based and NGS-based assemblers with LR_Gapcloser to produce high-quality assemblies.

## Findings

### The algorithm of LR_Gapcloser

**Figure 1:**
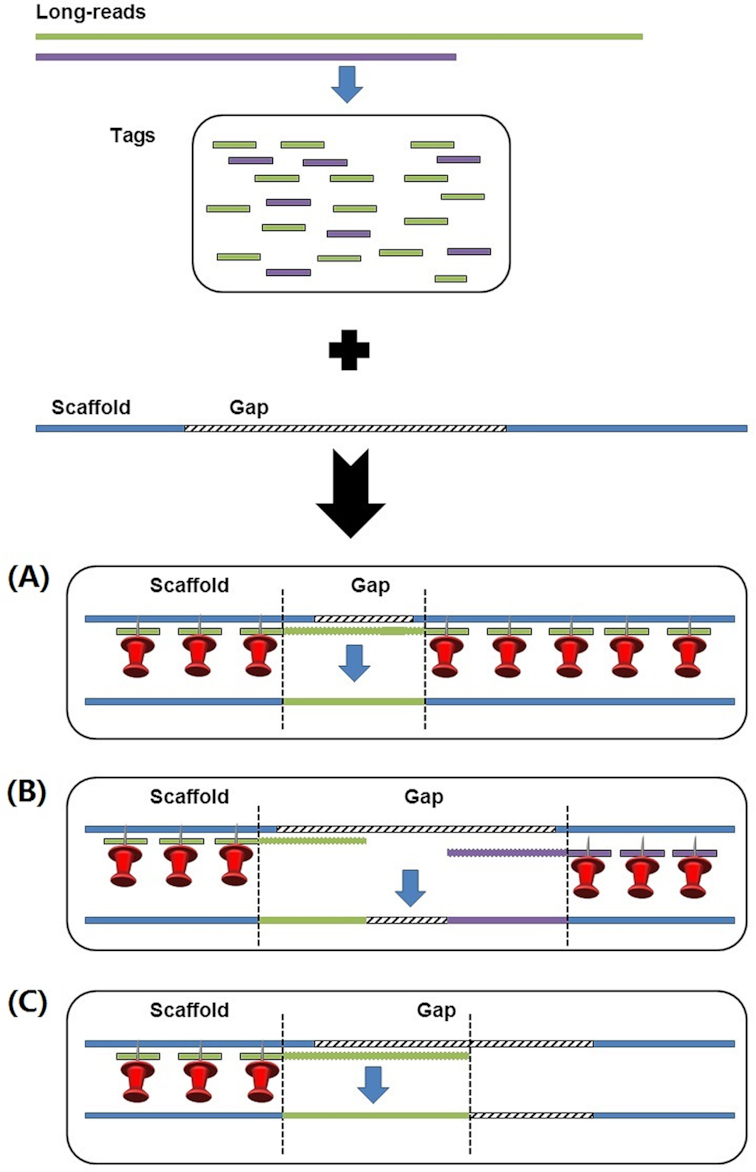
The primary steps in LR_Gapcloser. The long reads are colored green and purple. The long reads are fragmented into tags of the same length. The scaffolds are colored blue and the gaps marked as skew lines. The tracks represent that the tags are aligned to the scaffolds. **(A)** A gap that is completely closed with a long read. **(B)** A gap that is filled with two reads. **(C)** A gap that could be partially closed by only one read.

The primary steps of the LR_Gapcloser algorithm are outlined in Fig. 1. This tool can use either uncorrected or error-corrected long reads to fill gaps.

#### Fragmentation of long reads to short tags in a tiling path

Each long read was fragmented into short tags of the same length (default length: 300 bp). All the tags were distributed in an orderly fashion along one long read without overlapping. The distribution of all the tags formed a tiling path along this read.

#### Alignment and filtration

All the tags were aligned to genome sequences using the BWA-MEM (Burrows-Wheeler Aligner Maximal Exact Match) algorithm [21]. We calculated the coverage of each alignment based on the following formula:

\begin{equation*} {\mathrm{Coverage}} = \frac{{\left( {match\ base\ length + insert\ base\ length} \right)}}{{tag\ length}}
\end{equation*}

We adopted the definitions of the match base and the insert base as described in the SAM (Sequence Alignment/Map) format specification [22]. The match base length is the sum of identical bases between one read and its aligned sequence. The insert base length is the total number of bases inserted into the reference genome sequence. If the coverages of the alignments were over a certain threshold (default coverage of 80%), then these alignments were retained. We classified the tags into the following two types: tags uniquely aligned to one genomic locus and tags having at least two aligned positions. We performed three rounds of filtrations.

For the tags of type 1, we employed the following strategies to refine the orientations and orders of uniquely aligned tags. First, the orientations of uniquely aligned tags in one scaffold possibly conflicted with one another due to the sequencing error in the long read or the misassemblies in this scaffold. Based on the alignments of the tags of type 1, we calculated the tag numbers of each orientation. If the tag number from each alignment orientation was equal, then all the alignments from these reads to this scaffold were potentially error-prone and thus removed. If one alignment orientation had more uniquely aligned tags than the other orientation, then all alignments of the former orientation were retained and alignments of the latter orientation were removed. Second, the order of uniquely aligned tags in one read might not be consistent with the order in the corresponding scaffold due to misalignment or misassembly. If a read had only two retained tags and the order of these two tags in one read were consistent with the order in the scaffold, these tags were retained; otherwise, these tags were removed. If a read had more than two retained tags, then we selected the tag at the median position on the read and its position on the scaffold as reference. We compared the positions of the other tags with the position of the reference tag. If the order of the compared tag and the reference tag on the read were consistent with the order on the scaffold, then this compared tag and its alignment were retained; otherwise, it was removed.

After we refined the alignment of the tags of type 1, we searched the best alignment for each tag of type 2 using the following two criteria: the aligned scaffold and corresponding orientation should be the same as that of the neighboring tags of type 1 and the relative position of this tag compared to the neighboring tags of type 1 on the read should be consistent with the one on the scaffold.

#### Selection of supporting reads and gap closure

We tallied the gaps into the following three types: (1) two boundaries of one gap had aligned tags from one read. The tag number at each boundary should be over a threshold (default number of 5) to ensure accurate alignments. Tags at one boundary formed pairs with tags at the other boundary. Assuming that two tags, *a* and *b* from read (A) were aligned to two boundaries of a gap (B), then these two tags formed a pair. We retained this pair in the downstream analysis if it satisfied the following condition:

\begin{equation*} 1 - {\boldsymbol{c}} < \frac{{p({\boldsymbol{b}}) - p({\boldsymbol{a}})}}{{O({\boldsymbol{b}}) - O({\boldsymbol{a}})}} < 1 + {\boldsymbol{c}}
\end{equation*}

where p(*a*) and p(*b*) were the positions in read (A), O(*a*) and O(*b*) were the positions in the aligned scaffold, and the variable *c* was the allowed length derivation between the distance in the read and the one in the scaffold (default setting as 0.2).

The retained tag pair was then used as supporting evidence for this read to close the gap. For each gap, the read with the most tag pairs was selected, and the sequence between the two tags nearest to the two boundaries of the gap was used to fill the gap.

(2) Two boundaries of one gap had multiple tag alignments from different reads. At each boundary, if the read had more than a minimum number (default number of 5) of aligned tags and it covered the largest proportion of the gap, this read was selected. Two reads were retained to fill the gap. For each read, we determined the tag nearest to the gap, and the sequence from this tag to the end of the read was used to fill the gap.

We assumed that a tag (*a*) from read (A) and another tag (*b*) from read (B) were aligned nearest to each boundary of gap (C). The lengths of read (A), read (B), and gap (C) were L(A), L(B), and L(C), respectively. The position of tag (*a*) in read (A) was p(*a*) and the position of tag (*b*) in read (B) was p(*b*). If the sum of the bases closed by these two reads was smaller than 100%, i.e.,

\begin{equation*} {\mathrm{L}}\left( {\boldsymbol{A}} \right) - {\mathrm{p}}\left( {\boldsymbol{a}} \right) + {\mathrm{p}}\left( {\boldsymbol{b}} \right) < L\left( {{\boldsymbol{C}}\ } \right), \end{equation*}

then this gap was not completely closed. A sequence composed of the letter “N” was inserted between two filled sequences. The number of inserted “N”s was the difference between the gap length and the length of the filled sequence, where

\begin{equation*} {\mathrm{N}} = {\mathrm{L}}( {\boldsymbol{C}} ) - ( {L( {\boldsymbol{A}} ) - p( {\boldsymbol{a}} ) + p( {\boldsymbol{b}} )} ). \end{equation*}

If the sum of bases closed by these two reads was larger than or equal to 100%, i.e.,

\begin{equation*} L({\boldsymbol{A}}) - p({\boldsymbol{a}}) + p({\boldsymbol{b}}) \geq {L}( {\boldsymbol{C}} ). \end{equation*}

then the sequence from p(*a*) to the end of read (A) was retrieved. The sequence from the start of read (B) to p(*b*) was retrieved and connected to the above sequence to fill the gap. One hundred “N”s were inserted between two filled sequences to indicate a gap that could possibly be completed.

(3) Only one boundary of one gap had multiple tag alignments from different reads. The read covering the largest proportion of the gap was selected. After the tag nearest to the gap was determined, the sequence from it to the end of the read was used to fill the gap. We inserted a sequence composed of the letter “N.” The number of inserted “N”s was the difference between the gap length and the length of the filled sequence.

The entire gap-closing process can be iteratively implemented. Gaps in the types described in (2) and (3) would be further completed as the type described in (1) following the subsequent next iteration of gap closure.

### Closure of gaps in NGS-generated assemblies

All public sequencing reads used in this study are described in Additional file 1 (Tables S1, S2, and S3; see the section “Sequencing read sets and selected reference genomes” below). We first examined the performance of each tool (LR_Gapcloser, PBJelly [18], and GMcloser [15]) on NGS assemblies of *Saccharomyces cerevisiae* S288C, *Caenorhabditis elegans* Bristol, and human CHM1 chromosome X (HsX hereafter) using the raw Pacbio reads. The raw reads were not corrected and were directly fed into these three tools. For *S. cerevisiae*, which has a small genome, the contig N50 of the NGS assembly was 43.4 kb (Table 1). It included 421 gaps with a total length of 290.8 kb and a maximal gap length of 13.5 kb (Additional File 1, Table S4). Compared against the reference assembly, the input assembly included 29 misassemblies and covered 5,958 complete genes. LR_Gapcloser closed almost all gaps and filled the most bases (99.97%; Additional file 1, Table S4; Additional file 2, Table S5) at the lowest error rate (the ratio of misassemblies to contig N50, 3.8 × 10^−5^; Table 1). LR_Gapcloser led to the greatest increase in the contig N50 (18.1 fold) and had a runtime of 24 minutes, approximately 1.6% of the runtime of GMcloser (the second-fastest tool using 24 hours 30 minutes; Additional file 1, Table S4). The peak memory usage of LR_Gapcloser was only 3.8% of that by GMcloser and 18.6% of that by PBJelly.

**Table 1:** Gap-closure performance in the NGS-generated assemblies with PacBio reads, for contigs ≥1,000 bp

Reads	Tool	No. of contigs	Contig N50 (bp)	Contig NGA50 (bp)	Total length without gaps (bp)	Genome fraction (%)	Misassemblies	Ratio of misassemblies to N50 (× 10^−5^)	Ratio of misassemblies to NGA50 (× 10^−5^)	No. of complete genes
*S. cerevisiae* S288C										
	Initial assembly	472	43,406	37,861	11,275,980	92.965	29	66.8	76.5	5,958
Uncorrected PacBio reads	GMcloser	472	43,406	37,861	11,275,980	92.965	29	66.8	76.5	5,958
	PBJelly	228	129 ,334	97,464	12,123,886	96.044	102	78.9	104.6	6,185
	LR_Gapcloser	70	784,212	310,248	11,603,845	94.653	30	3.8	9.7	6,115
Corrected PacBio reads	GMcloser	100	356,432	213,603	11,540,046	94.563	37	10.3	17.3	6,107
	PBJelly	205	126,144	98,570	11,761,355	95.733	48	38.0	48.6	6,190
	LR_Gapcloser	75	706,777	296,575	11,586,142	95.016	35	4.95	11.8	6,136
*C. elegans* Bristol										
	Initial assembly	4,256	43,175	40,534	95,538,867	95.587	73	169.1	180.1	42,116
Uncorrected PacBio reads	GMcloser	4,256	43,175	39,576	95,538,867	95.587	157	363.6	396.7	42,116
	PBJelly	1,968	123,707	85,754	102,625,904	98.951	1,480	1196.4	1725.9	43,415
	LR_Gapcloser	341	2,603,168	443,773	99,631,648	98.364	390	15.0	87.9	43,841
Corrected PacBio reads	GMcloser	657	537,136	298,230	99,579,391	97.731	424	78.9	142.2	43,674
	PBJelly	2,017	111,801	85,058	100,911,552	98.576	1,018	910.5	1196.8	43,339
	LR_Gapcloser	402	1,570,967	498,533	99,476,660	98.159	291	18.5	58.4	43,748
*H. sapiens* CHM1 chromosome X										
	Initial assembly	3,433	76,525	74,285	145,883,728	97.657	116	151.6	156.2	386
Uncorrected PacBio reads	GMcloser	3,433	76,525	74,285	145,883,728	97.657	116	151.6	156.2	386
	PBJelly	1,280	262,085	226,918	150,211,820	99.276	470	179.3	207.1	482
	LR_Gapcloser	297	2,606,228	649,184	149,690,339	98.606	470	18.0	72.4	543
Corrected PacBio reads	GMcloser	1,427	236,652	214,405	147,443,419	98.471	248	104.8	115.7	492
	PBJelly	1,490	211,029	190,153	149,938,882	99.077	608	288.1	319.7	472
	LR_Gapcloser	556	773,990	440,314	148,084,716	98.790	486	62.8	110.4	544

For *C. elegans*, a medium-sized genome, the contig N50 of the NGS assembly was 43.2 kb. This assembly had 4,452 gaps, which accounted for 3.67 Mb, with a maximal length of 12.5 kb. It included 73 misassemblies and covered 42,116 complete genes (Table 1). LR_Gapcloser also exhibited the best performance among the three tools. After gap-closure by LR_Gapcloser, at the lowest error rate (the ratio of misassemblies to contig N50, 15 × 10^−5^), the most gap bases (99.7%) were filled and the most gaps (96.4%) were closed, leading to the greatest increase in the contig N50 (60.3 fold) and the greatest decrease in the contig number (Table 1; Additional file 1, Table S4; Additional file 2, Table S5). The assembly closed by LR_Gapcloser covered the highest number of complete genes (43,841). Furthermore, LR_Gapcloser had the shortest runtime (4 hours and 25 minutes), 7.5% of the runtime of GMcloser (the second-fastest tool, running over 59 hours; Additional file 1, Table S4). In addition, the peak memory usage of LR_Gapcloser was much lower than that of the other two tools.

A similar tendency was observed for the HsX assembly. The contig N50 of the NGS assembly was 76.5 kb. It included 3,737 gaps with a total length of 2.05 Mb. With the most closed gaps (94.4%), LR_Gapcloser added the most nucleotides (98.8%) to gaps at the lowest error rate (the ratio of misassemblies to contig N50, 18 × 10^−5^; Table 1; Additional file 1. Table S4; Additional file 2, Table S5). This tool produced the greatest increase of the contig N50 (34.1 fold). The runtime of LR_Gapcloser was the shortest (3 hours and 53 minutes), at approximately 32.6% of the runtime of PBJelly (the second-fastest tool with a runtime of approximately 12 hours; Additional file 1, Table S4). The comparison on the NGS-produced gaps demonstrated that the efficiency, accuracy, and speed of LR_Gapcloser were considerably higher than those of the other tools. These results also suggested that for assemblies of small contigs, the contig N50 size could be significantly improved.

We then tested the performance of each tool on these three NGS assemblies using the error-corrected PacBio reads, and demonstrated that among the three tools, LR_Gapcloser closed the most gaps at the lowest error rate. LR_Gapcloser required a shorter runtime and lower peak memory than the other two tools (Table 1; Additional file 1, Table S4). A similar fast and efficient performance of LR_Gapcloser was also observed when closing gaps in the NGS-generated assemblies of *S. cerevisiae* and *C. elegans* using either the raw Nanopore reads or error-corrected Nanopore reads (Additional file 1, Tables S6 and S7; Additional file 3, Table S8).

### Closure of repeat-derived gaps

Due to their high similarity, repetitive regions are difficult to close, creating a challenge to complete genome assembly. We evaluated the closure performances of the above three tools on the repeat-derived gaps using the raw PacBio reads, the error-corrected PacBio reads, the raw Nanopore reads, and the error-corrected Nanopore reads. We first tested the performance of each tool using the raw PacBio reads. The raw reads were not corrected and directly used as input for the three tools. In the *S. cerevisiae* assembly, we designated 464 repeat-derived gaps, the total size of which was 513.4 kb (Table 2; Additional file 1, Table S9). The gap sizes ranged from 200 bp to 11,510 bp. These gaps broke the reference genome into an error-free contig set including 299 contigs, in which the contig N50 size was 77.84 kb. The new assembly generated by GMcloser was 20.7 Mb, much larger than the reference genome size (12.1 Mb), indicative misclosures caused by GMcloser (Additional file 4, Table S10). The runtime of LR_Gapcloser was 44 minutes, only 2.9% of PBJelly runtime (Additional file 1, Table S9). The peak memory usage of LR_Gapcloser was also the lowest, 13.5% of the usage required by PBJelly. LR_Gapcloser filled the most gap bases with 99.4% and finished almost all gaps (except nine unclosed gaps) at the lowest error rate (the ratio of misassemblies to contig N50, 0.98 × 10^−5^; Additional file 1, Table S9). This tool led to the greatest increase in the contig N50 (10.4 fold) with the highest number of complete genes (6,403) (Additional file 4, Table S10).

**Table 2:** Closure on repeat-derived gaps in the reference assemblies with PacBio reads, for contigs ≥1,000 bp

Reads	Tool	No. of contigs	Contig N50 (bp)	Contig NGA50 (bp)	Total length without gaps (bp)	Genome fraction (%)	Misassemblies	Ratio of misassemblies to N50 (× 10^−5^)	Ratio of misassemblies to NGA50 (× 10^−5^)	No. of complete genes
*S. cerevisiae* S288C										
	Initial assembly	299	77,840	76,116	11,598,097	95.584	0	0	0	6,190
Uncorrected PacBio reads	GMcloser	502	79,876	120,645	20,723,339^a^	95.584	0	0	0	6,190
	PBJelly	67	560,335	475,256	12,629,537	99.569	51	9.1	10.7	6,390
	LR_Gapcloser	22	813,198	781,485	12,153,365	99.795	8	0.98	1	6,403
Corrected PacBio reads	GMcloser	108	553,180	811,382	19,143,529^a^	99.379	13	2.35	1.6	6,388
	PBJelly	98	317,546	297,528	12,268,477	98.896	32	10.1	10.8	6,343
	LR_Gapcloser	41	666,801	555,815	12,136,999	99.684	4	0.6	0.72	6,396
*C. elegans* Bristol										
	Initial assembly	406	510,819	508,558	99,305,410	99.033	0	0	0	44,678
Uncorrected PacBio reads	GMcloser	406	510,819	508,558	99,305,410	99.033	0	0	0	44,678
	PBJelly	80	2,517 ,082	991,194	100,899,157	98.705	308	12.2	31.1	44,393
	LR_Gapcloser	23	10,361,459	4,731,490	100,224,225	99.687	36	0.35	0.76	44,794
Corrected PacBio reads	GMcloser	152	1,075,373	846,874	99,962,602	99.54	70	6.51	8.27	44,778
	PBJelly	125	1,585,684	892,872	100,298,205	99.619	136	8.58	15.2	44,782
	LR_Gapcloser	51	4,509,353	2,055,178	100,136,981	99.696	61	1.35	2.97	44,792
*H. sapiens* CHM1 chromosome X										
	Initial assembly	977	289,166	271,924	146,253,194	97.706	0	0	0	719
Uncorrected PacBio reads	GMcloser	977	289,166	27,1924	146,253,194	97.706	0	0	0	719
	PBJelly	33	14,496,807	5,459,128	149,735,973	99.917	65	0.49	1.19	791
	LR_Gapcloser	12	16,029,035	8,219,635	149,673,140	99.945	50	0.31	0.61	789
Corrected PacBio reads	GMcloser	444	718,976	715,918	148,447,825	99.090	8	1.11	1.12	754
	PBJelly	157	1,558,141	1,398,081	149,647,694	99.726	86	5.52	6.15	777
	LR_Gapcloser	39	6,778,534	4,573,022	149,621,524	99.930	45	0.66	0.98	788

a The assembly size after gap closure is much larger than the reference genome size.

In the *C. elegans* assembly, 480 repeat-derived gaps were created with a total size of 964.7 kb (Additional file 1, Table S9). The error-free set included 406 contigs longer than 1 kb. The contig N50 size was 510.8 kb (Table 2). In the new assembly by LR_Gapcloser, in total 92.5% of gap bases were filled (Additional file 1, Table S9), resulting in 92.5% finished gaps (Additional file 1, Table S10). The contig N50 was increased to 10.4 Mb with a 20.3-fold improvement. The complete gene number was the highest (44,794; Additional file 4, Table S10) and the ratio of misassemblies to contig N50 was the lowest (0.35 × 10^−5^). LR_Gapcloser was also faster and had lower peak memory usage than the other two tools (Additional file 1, Table S9).

We produced 1,044 repeat-derived gaps in the HsX assembly, the total size of which was 3.43 Mb. The error-free set consisted of 977 contigs over 1 kb. The similar fastest and best performance of LR_Gapcloser was observed in this comparison to the other two tools. Our tool added the most nucleotides to the gap regions (99.6%) at the lowest error rate (the ratio of misassemblies to contig N50: 0.31 × 10^−5^). Over 94% of gaps were finished. It had the shortest runtime of 5 hours and 33 minutes (Additional file 1, Table S9). The contig N50 size increased from 289.2 kb to 16 Mb (a 55.4-fold increase). The gap-closed assembly covered the highest proportion of the reference genome (99.95%, Table 2).

Comparing the performances of three tools on these repeat-derived gaps using the error-corrected PacBio reads, the raw Nanopore reads, and the error-corrected Nanopore reads further demonstrated that among the three tools, LR_Gapcloser closed the most gaps at the lowest error rate with the shortest runtime and lowest peak memory (Table 2; Additional file 1, Tables S11 and S12; Additional file 2, Table S13). These results also support that our tool could be an efficient solution for closing gaps in repetitive regions.

### Closure of real gaps

We further tested the closure performances of the three tools on real gaps in the HsX assembly using either the raw PacBio reads or error-corrected reads. Although 2,279 contigs were anchored to the HsX sequence (155 Mb) using a reference-guide assembly strategy [23], the contig N50 size was only 157 kb. The longest gap located at the centromere was 3 Mb. The total size of the other 2,278 gaps accounted for 2.49 Mb. When using the raw PacBio reads, although LR_Gapcloser had a longer runtime than PBJelly, LR_Gapcloser made the greatest increase of contig N50 from 157 kb to 4.07 Mb and decreased the gap number from 2,280 to 166 (Additional file 1, Table S14). When using the error-corrected PacBio reads, we observed the same optimal performance of LR_Gapcloser.

To test the scalability and performance of the tools in closing real gaps with Nanopore data, we closed the gaps in the largest scaffold (KQ079791) of human GM12878 assembly using these three tools. This scaffold had 53 gaps and the contig N50 size was 2.86 Mb. We found that when using either the raw Nanopore reads or error-corrected Nanopore reads, LR_Gapcloser closed the most gaps, had the shortest runtime, and required the lowest peak memory (Additional file 1, Table S15).

### Improving the contiguity of the reference-quality assemblies by different approaches and from large and complex genomes

Overall, the above three comparisons on NGS-generated gaps, repeat-derived gaps, and real gaps revealed that LR_Gapcloser closed more gaps with remarkably smaller error rates than the other methods. In addition to mate pair-based scaffolding [24], which was commonly used in genome projects, other assembly strategies have been used for genome assembly and they can produce reference-quality assemblies with much longer contigs. These strategies include reference-guide assembly [25], the third generation of the single-molecule sequencing strategy [26], and next-generation mapping [27]. These strategies generated reference-quality assemblies with a much higher scaffold N50 size, although there were still gaps. Thus, we assessed the performance of LR_Gapcloser in improving the contiguity of the reference-quality genome assemblies by a range of approaches.

The reference CHM1 genome assembly included 40,893 contigs, which were anchored to 23 chromosomes with a reference guide strategy [23]. The assembly included 40,915 gaps (a total length of 210.2 Mb). The contig N50 size was 143.9 kb with the longest contig size of 7.16 Mb (Table 3). Using the raw PacBio reads, LR_Gapcloser incorporated novel nucleotides of 30.2 Mb into the assembly. The number of gaps and contigs decreased to 522 and 508, respectively. Notably, the contig N50 size increased to 19.08 Mb and the largest contig increased to 57.1 Mb. The updated contig N50 size was larger than that generated by MHAP (4.32 Mb) [26] and MECAT (4.88 Mb) [28], both of which are TGS-based *de novo* assemblers.

**Table 3:** Improvements of three reference-quality human assemblies

Accession	Status	Gap number	Contig number	Total length without gaps (bp)	Total gap length (Mb)	Contig N50 (bp)	Largest contig (bp)
GCA_000306695 (CHM1 genome)	Before gap closure	40,915	40 893	2,827,653,301	210.23	143,921	7,163,879
	After gap closure	522	508	2,857,813,755	180.04	19,078,895	57,098,690
GCA_001013985 (GM12878 cell genome)	Before gap closure	2,332	21,235	3,030,222,093	146.35	1,557,716	10,883,701
	After gap closure	210	19,113	3,126,235,473	50.67	12,989,464	49,206,979
GCA_001750385 (Korean genome)	Before gap closure	264	3,096	2,866,867,749	37.34	18,080,262	76,477,139
	After gap closure	112	2,944	2,888,283,666	15.98	23,374,317	85,176,535

The GM12878 cell genome assembly was generated by combining single-molecule sequencing reads with single-molecule genome maps [29]. Although the scaffold N50 value was over 26.8 Mb, the contig N50 was 1.56 Mb and the total gap size was 146.4 Mb. Using the raw PacBio reads, LR_Gapcloser incorporated novel nucleotides of 95.7 Mb into the assembly. The gap number was reduced by 91%. Remarkably, the contig N50 size increased from 1.56 Mb to 12.99 Mb, and the largest contig was extended from 10.9 Mb to 49.2 Mb (Table 3).

PacBio sequencing, BioNano mapping, linked reads, and bacterial artificial chromosome (BAC) sequencing approaches were integrated to produce a high-quality Korean human genome assembly [30]. The high contiguity was represented by a contig N50 size of 18.1 Mb and a total gap size of 37.3 Mb. Our tool added 21.4 Mb (57.3%) of novel nucleotides to this assembly. The contig N50 size increased to 23.4 Mb (Table 3) and the longest contig was extended to 85.2 Mb. These applications suggested that LR_Gapcloser was suitable for the contiguity improvement of genome assemblies generated by different strategies, even in high-quality assemblies.

We demonstrated the efficient performance of LR_Gapcloser when applied to large genomes as humans. The scalability of this tool to close gaps in larger genome assemblies was further examined by using it to close the gaps in the *Triticum urartu* genome, the progenitor of wheat A subgenome [31]. This genome is 4.94 Gb in size and repeat rich. Although some gaps were closed in this genome by using PBJelly, there was still a total gap size of 30 Mb. The contig N50 size and longest contig size were 278.4 kb and 2.07 Mb, respectively. Our tool added 11 Mb (36.7%) of novel nucleotides to the gap regions. The contig N50 size and longest contig size increased to 389.3 kb and 3.67 Mb, respectively. These data demonstrated that this method is applicable on the small-, medium-, and large-sized genomic spectrum.

### A proposed hybrid assembly strategy using TGS and NGS reads

The TGS-based assembly exhibits high contiguity, represented by a large contig N50 value [32]. The advantage of NGS-based assembly is represented by a large scaffold N50 value. For species in which both NGS and TGS reads were generated, we posed a question how to integrate the assembly, scaffolding, and gap closure to generate a reference-grade genome assembly. There are currently six assembly strategies that have been developed to combine the NGS and TGS reads (see “Performance comparison of different hybrid assembly strategies by using TGS and NGS reads” below). We applied these strategies to assembly of the genomes of *S. cerevisiae, C. elegans*, and HsX and evaluated the contiguity and correctness of each strategy.

**Figure 2:**
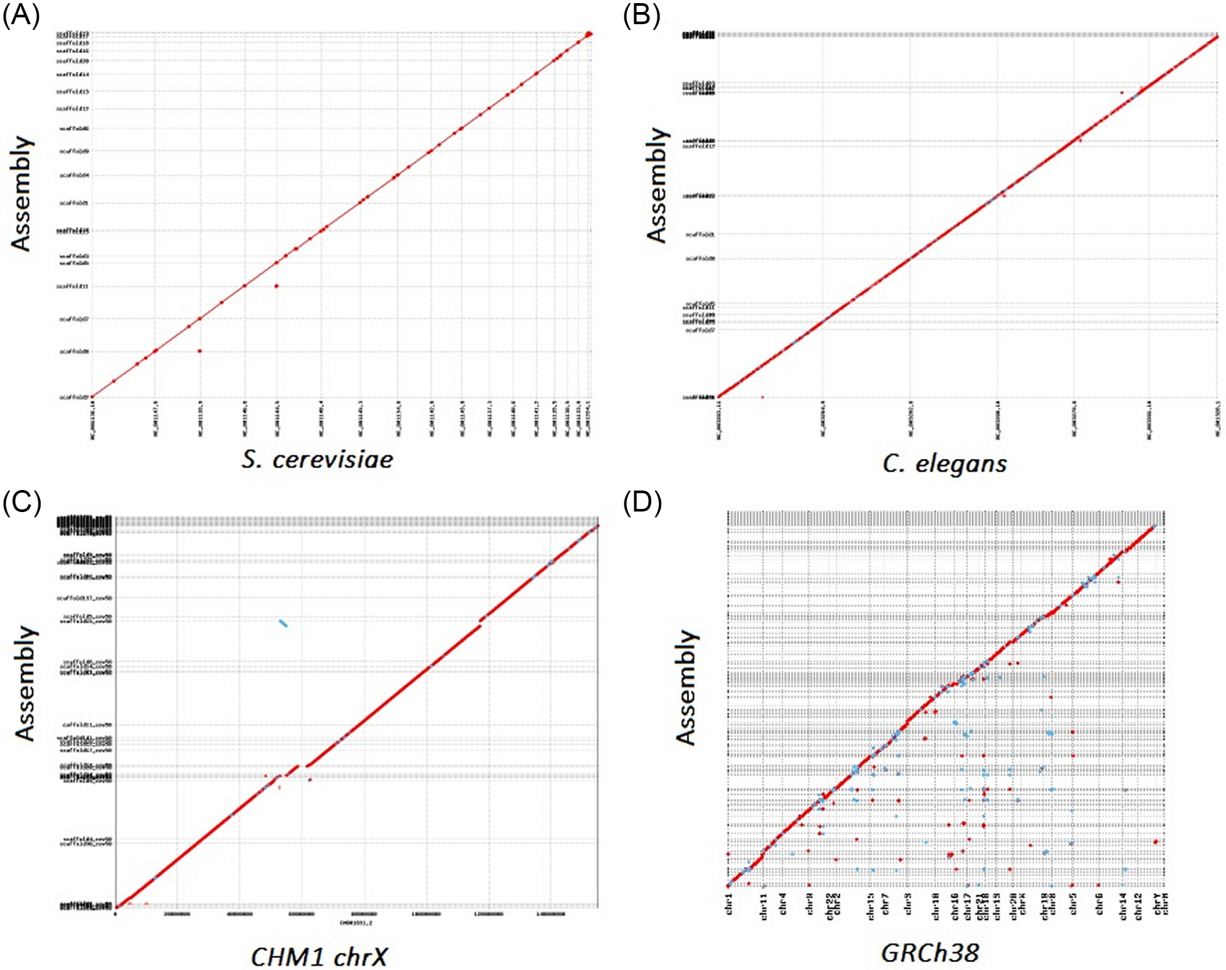
MUMmerplot of new assemblies generated by strategy 5 compared to the reference assemblies. Alignment dot plots show the structural agreements between the new assemblies (*y*-axis) and the reference (*x*-axis). Boundaries of chromosome from the reference and of scaffolds from the assemblies are represented as dotted lines (vertical and horizontal, respectively). Scaffolds are oriented and ordered to match the chromosomes using “mummerplot” command with “–filter –layout” option. **(A)** Plot between the new assembly and *S. cerevisiae* reference. **(B)** Plot between the new assembly and *C. elegans* reference. **(C)** Plot between the new assembly and CHM1 HsX reference. **(D)** Plot between the new CHM1 assembly and GRCh38 reference.

In *S. cerevisiae*, the assemblies produced by the strategies with the TGS-based assembler (strategy 3, 4, and 5) had higher genome coverages, higher contiguity, and lower error rates than those produced with the NGS-based assembler (strategy 1 and 2) and hybridSPAdes [33] (strategy 6). Although the misassembly number and the ratio of misassemblies to contig N50 (NGA50) in the assembly by strategy 5 ranked third, which were slightly lower than those by strategy 3 and 4, all the other metrics, including the coverage and contiguity of this assembly, ranked first. The genome coverage of this assembly reached 99.5%. The sequence identity compared to the reference genome was as low as 5.23 mismatches and 18.78 insertions and deletions (indels) per 100 kb (corresponding to 99.97% accuracy) (Additional file 4, Table S16). The gaps in all 20 scaffolds of the new assembly generated by strategy 5 were completely closed, leading to a scaffold N50 value of 834.2 kb and a contig N50 value of 834.2 kb (Table 4). The assembly completed 14 out of 17 chromosomes (Fig. 2A). The other three chromosomes were spanned by just two contigs. These results reveal the perfect continuity of the assembly under strategy 5.

**Table 4:** Comparison of assemblies generated by different hybrid assembly strategies for sequence ≥1000 bp

Strategy	No. of scaffolds	Scaffold N50 (bp)	No. of contigs	Contig N50 (bp)	Contig NGA50 (bp)	Total length (bp)	Genome fraction (%)	Mis-assemblies	Ratio of misassemblies to contig N50 (× 10^−5^)	Ratio of misassemblies to NGA50 (× 10^−5^)	No. of complete genes
*S. cerevisiae* S288C											
Strategy 1	70	782,307	472	43,406	37,861	11,275,980	92.965	29	66.8	76.6	5,958
Strategy 2	70	784,212	70	784,212	310,248	11,603,845	94.653	30	3.8	9.7	6,115
Strategy 3	NA	NA	26	752,311	548,251	12,242,107	99.504	22	2.92	4.01	6,381
Strategy 4	20	834,245	25	751,743	548,251	12,232,978	99.498	21	2.79	3.83	6,378
Strategy 5	20	834,245	20	834,245	553,541	12,241,279	99.504	27	3.24	4.88	6,382
Strategy 6	81	735,436	134	286,930	188,069	11,679,475	95.82	33	11.5	17.5	6,121
*C. elegans* Bristol											
Strategy 1	317	7,522,240	4,256	43,175	40,534	95,538,867	95.587	73	169.1	180.1	42,116
Strategy 2	317	7,540,932	341	2,603,168	443,773	99,631,648	98.364	390	15.0	87.9	43,841
Strategy 3	NA	NA	195	1,207,434	381,285	101,862,626	97.363	586	48.5	153.7	43,709
Strategy 4	83	12,886,407	194	1,367,460	407,816	101,775,561	99.119	541	39.6	132.6	44,214
Strategy 5	83	12,886,574	83	12,886,574	433,201	102,039,090	99.264	1,003	7.78	231.5	44,267
Strategy 6	3,939	803,810	5,452	83,934	69,291	100,356,139	94.838	597	711.2	861.6	41,613
*H. sapiens* CHM1 chromosome X											
Strategy 1	217	5,785,732	3,433	76,525	74,285	145,883,728	97.657	116	151.6	156.2	386
Strategy 2	217	5,787,692	297	2,606,228	649,184	149,690,339	98.606	470	18.0	72.4	543
Strategy 3	NA	NA	344	1,486,062	718,890	151,843,469	98.466	573	38.6	79.7	573
Strategy 4	141	15,922,371	338	1,486,062	718,778	151,744,013	98.451	572	38.5	79.6	573
Strategy 5	141	15,922,769	156	7,868,846	970,260	152,338,003	98.750	669	8.5	68.9	592
Strategy6	Terminated, cause unclear										

In *C. elegans*, we found that the assemblies solely relying on either TGS reads or NGS reads (strategies 1 and 3) had lower contig N50 sizes than the assemblies using the step-wise hybrid assembly of TGS and NGS reads (strategies 2, 4, and 5). Apart from the ratio of misassemblies to NGA50, all other metrics of this assembly by strategy 5 ranked first. This assembly consisted of 83 scaffolds and 83 contigs without any gaps and had the highest scaffold N50 value (12.89 Mb) and contig N50 value (12.89 Mb; Table 4). The genome coverage reached 99.26%. The sequence identity compared to the reference genome was higher than 99.93% (14.26 mismatches and 56.72 indels per 100 kb) (Additional file 4, Table S16). The longest scaffold approached 18.67 Mb, covering 89.1% of chromosome V. Five autosomal chromosomes (V, IV, II, I, and III) were largely spanned by two scaffolds (Fig. 2B). The high coverage and contiguity with the low error rate demonstrated that strategy 5, along with a sufficient coverage of long reads and short paired-end reads, could generate *de novo*-assembled genomes that approach reference quality.

We also observed the best performance of strategy 5 in the HsX assembly. This assembly had the best value for all the metrics, except for the genome coverage (98.75%). Despite the original CHM1 Genome Project, including deep-coverage Illumina reads and BAC clones, the contig N50 of the new HsX assembly was greater than that of the reference genome size (7.87 Mb vs 157 kb) with fewer contigs (156 vs 2279). Compared with the reference HsX assembly, the new assembly had an accuracy of 99.83% (13.22 mismatches and 156.98 indels per 100 kb) (Additional file 4, Table S16). Furthermore, the new HsX assembly was structurally consistent with the reference assembly (Fig. 2C).

Strategy 5, which used TGS-based and NGS-based assemblers and LR_Gapcloser, had the best performance and the lowest error rate to generate reference-grade assemblies. We applied this strategy to generating a new, highly contiguous CHM1 assembly. The new assembly consisted of 2,265 scaffolds with a scaffold N50 size of 28.45 Mb (Additional file 1, Table S17). It achieved marked contiguity with the largest contig N50 value (28.45 Mb) thus far (except the GRCh38 reference assembly). This value is an order of magnitude larger than the CHM1 reference assembly generated by the earlier method (143.9 kb) [23], and it is even larger than previous non-reference assemblies of the human diploid genome using long-range physical mapping strategies [29, 30, 34, 35] (Additional file 1, Table S17). To validate the assembly, according to the approach by Chaisson et al. [36], we compared 18 finished CHM1tert BAC clones [26, 36, 37] to the new assembly with MUMmer [20]. The assembly was structurally consistent with these BACs (Additional file 1, Fig. S1). Single-nucleotide polymorphisms identified with “show-snps” in the “dnadiff” [38] package revealed 99.96% shared identity (Additional file 1, Table S18). Comparing our assembly to the GRCh38 assembly showed a general agreement between both assemblies (Fig. 2D). These comparisons and validations suggest that the optimal and step-wise hybrid assembly strategy can generate the most contiguous genome assemblies for species in which both NGS and TGS reads were generated.

## Discussion

Compared with the existing two tools, here we summarize the advantages exhibited by our tool, LR_Gapcloser. First, our tool is fast and efficient with low memory usage. We designed three types of tests to compare the performances of the three tools. In all tests, LR_Gapcloser added the most nucleotides and closed the most gaps. In case of the ability to close NGS-generated gaps and repeat-derived gaps, LR_Gapcloser was 1.18~52.25 fold faster than PBJelly and 1.57~76.98 fold faster than GMcloser. In addition, the memory usage of LR_Gapcloser was the lowest, requiring only 3.2%~28.0% of the peak memory usage by GMcloser and 8.4%~32.9% of that by PBJelly. Second, LR_Gapcloser efficiently uses the raw long reads to close the gaps. When using the raw long reads, GMcloser fills few gaps, while LR_Gapcloser and PBJelly could utilize the raw reads to finish the gaps. However, LR_Gapcloser added more nucleotides than PBJelly. Third, LR_Gapcloser produced the fewest misassemblies. As shown in Tables 1 and 2, both the ratio of misassemblies to contig N50 and the ratio of misassemblies to contig NGA50 by LR_Gapcloser were much lower than that by the other two tools. Fourth, LR_Gapcloser has the best performance when applied to repeat-rich genomes. Repeats produce the greatest challenge to genome assembly. Tested on the repeat-derived gaps in the assemblies of different species, LR_Gapcloser closed at least 85.4% of the gaps, more than PBJelly and GMcloser. RepeatMasker [39] annotations of the added nucleotides to the real gaps in HsX by LR_Gapcloser indicate that more than 52% of the closed regions originate from repeats. Fifth, the fast and memory-efficient characteristics of LR_Gapcloser suggest that the present method is suitable for closing gaps in large assemblies. With 30 threads, PBJelly took 119 hours (Additional file 1, Table S4) with the uncorrected PacBio reads to finish the gaps in NGS-generated repeats in *C. elegans* (genome size of 0.1 Gb and gap size of 3.6 Mb). However, with 30 threads, LR_Gapcloser only required 63 hours to close the gaps in the *T. urartu* genome (genome size of 4.94 Gb and gap size of 30 Mb).

To achieve a rapid and optimal performance, we integrated three efficient approaches within LR_Gapcloser. First, it performs a strategy involving long read fragmentation and alignment. This strategy is 1.74~8.06-fold faster than the gap-closure tools that align the whole reads (Additional file 1, Fig. S2). Second, we constructed a pipeline to select the best-aligned reads for gap closure. PBJelly selects reads overlaying the gaps and performs consensus calling to finish the gaps. Without consensus base calling or assembly in the gap regions, the selection strategy used in LR_Gapcloser was much faster and generated fewer misassemblies than the consensus calling strategy. Third, many processes in LR_Gapcloser were parallelized in the gap-closing pipeline, including tag alignment to scaffolds, alignment coverage calculation, and filtration.

Over the last decade, the genomes of thousands of species have been sequenced using NGS technologies. The assemblies by NGS-based assemblers had large N50 sizes but small contig N50 sizes (Table 4). Long reads generated by TGS technologies provide promise for reconstructing complex genomic regions, including the repeat-rich heterochromatic regions of eukaryotic chromosomes. The hybrid and step-wise assembly of strategy 5 using the TGS and NGS reads could produce assemblies having both a high scaffold N50 value and a high contig N50 value (Table 4). In the best case, a reference chromosome was almost completely covered by one assembled contig. Our results showed that 14 *S. cerevisiae* chromosomes were resolved into single contigs. In addition to the high contiguity, this optimal strategy generates assemblies with comparable accuracy (e.g., 99.83% accuracy in a new HsX assembly) to those by NGS-based strategies (strategy 1, 99.99% accuracy) (Additional file 4, Table S16). To improve the base quality of the assemblies generated by this optimal strategy, the assemblies can be further polished with NGS data.

LR_Gapcloser is flexible in that both PacBio reads and Nanopore reads [40] can be utilized to fill the gaps. This tool is also likely to use the 10X genomic linked reads [41] and pre-assembled contigs to fill gaps in the assemblies. One limitation to the performance of LR_Gapcloser is that the current read length could not efficiently complete the extremely long gaps, especially in the centromeric and telomeric regions. Therefore, one future improvement is to generate new strategies to produce longer reads.

## Methods

### Sequencing read sets and selected reference genomes

We selected reads and reference genomes by implementing the following strict criteria: (1) the genome is complete or almost finished to facilitate the measurement of the gap-closure accuracy, and (2) PacBio long reads (or Nanopore long reads), NGS reads, and the reference genome assembly should be generated from the same strain to avoid misassemblies resulting from sequence variations between strains. The reference genomes of three species *(S. cerevisiae* S288C, *C. elegans* Bristol, and *H. sapiens* CHM1) are characterized by their different genome sizes and levels of complexity and were tested in this study. The reference genome assemblies and corresponding annotations of genes and repeats were taken from AsssemblyDB [42].

The raw Illumina sequencing reads were downloaded from the National Center for Biotechnology Information (NCBI) Sequence Read Archive (SRA) database (Additional file 1, Table S1). The reads were then trimmed using SolexaQA [43]. Pairs in which both high-quality ends were longer than 25 bp were retained. We also created random Illumina mate-paired reads with large insert sizes from the reference genome using DWGSIM [44] with the default parameters (Additional file 1, Table S1). For *S. cerevisiae*, one real paired-end library (insert size of 300 bp), one real mate-end library (insert size of 3,400 bp), and three simulated mate-end libraries (insert sizes of 5,000, 10,000 and 15,000 bp) with 570x genome coverage were input into the assembly. For *C. elegans*, this dataset included a real paired-end library (insert size of 250 bp) and four simulated mate-pair libraries (insert sizes of 3,000, 5,000, 10,000 and 15,000 bp). The sequencing coverage was over 380x. For *H. sapiens*, the dataset included one real paired-end library (insert size of 300 bp), two real mate-pair libraries (insert sizes of 3,000 and 8,000 bp), and two simulated mate-pair libraries (insert sizes of 10,000 and 15,000 bp).

The sources of all long reads used in this study are listed in Additional file 1, Tables S2 and S3. The raw reads (PacBio and Nanopore) for *S. cerevisiae* and *C. elegans* were obtained from the NCBI SRA database and other websites, respectively. MECAT [28] corrected the raw reads. Both the raw and error-corrected reads were employed into the comparison of the gap-closure performances.

For a gap-closure comparison of the human CHM1 genome with PacBio reads, we used only chromosome X (HsX) as a representative for the whole genome. We selected this small chromosome, 1/20 the size of the genome (155 Mb) because the existing gap-closers would take many days and require high memory usage to finish the gaps of the CHM1 genome (NCBI AssemblyDB: GCA_000306695). The Illumina reads were mapped against the entire genome using the BWA-MEM algorithm in the BWA package [45]. If at least one read on one Illumina pair was mapped to HsX, then this pair was employed in the *de novo* assembly of HsX. We also mapped the raw PacBio reads to the reference genome using BWA-MEM (with the parameter of –x pacbio) and extracted the long reads mapped to HsX. The mapped raw reads were corrected using MECAT. Both the raw and error-corrected PacBio reads were input into the comparison of the closure performances on the NGS-generated gaps, repeat-derived gaps, and real gaps.

Further, since there are no available Nanopore reads from the human CHM1 genome to compare the closure performances of different tools on real gaps with Nanopore reads, the Nanopore reads from human GM12878 genome were used (NCBI AssemblyDB: GCA_001013985) [46]. Following the above strategy, we selected the large scaffold (NCBI Accession: KQ079791) to represent the whole genome, with a length of 80.3 Mb. All raw Nanopore reads were first mapped against the GM12878 genome assembly using the BWA-MEM algorithm (with the parameter of -x ont2d). The raw reads mapped to this scaffold were then corrected using MECAT. The raw and error-corrected Nanopore reads were used to close gaps.

### Evaluation of gap-closure performance by three tools

We compared the gap-closure performance of LR_Gapcloser and two currently available tools, GMcloser [15] and PBJelly [18]. These two tools were run using the default parameters. We ran each tool with 30 threads on the same machine. We estimated the performance of each tool using following six indicators: closed gap number, filled base number, runtime, peak memory usage, quality metrics by Quast [47], and misassembly ratio.

For each tool, the closed gap number was the difference between the gap numbers in the two assemblies before and after gap closure. The filled base number was the difference between the total gap length in the closed assembly and the one in the input assembly. To evaluate the gap-closure accuracy, each assembly was split into contigs, which were compared against the reference genome using Quast [47] with the parameters of –min-alignment 500 and –min-identity 90. To evaluate the quality of the genome assembly, Quast introduces a series of quality indicators that are grouped into four categories: (i) contig sizes, (ii) misassemblies and structural variations, (iii) genome representation and its functional elements, and (iv) variations in N50 based on aligned blocks. Quast defines one misassembly in one contig as a position where: (i) the left flanking sequence and the right flanking sequence are over 1 kb apart on the same reference sequence, or (ii) these two flanking sequences have an overlapping region over 1 kb, or (iii) they align on opposite strands of the same genomic sequence, or (iv) two different chromosomes [47]. The misassemblies were further classified by Quast into three groups, including relocation (i and ii), inversion (iii), and translocation (iv).

It is reasonable that more misassemblies were generated as more gaps were finished and the contig N50 size increased, thus we introduced two ratios to normalize the misassemblies:

- *Ratio of misassemblies to contig N50*: The total number of misassemblies divided by the contig N50 size.
- *Ratio of misassemblies to NGA50*: The total number of misassemblies divided by the NGA50 size. The contigs in the new assemblies are split into aligned blocks and the NGA50 is the NG50 of the aligned blocks.

### Comparison of the performances of three tools on gaps of three types from different species

We compared the gap-closure performances of the three tools on three types of gaps: NGS-generated gaps, repeat-derived gaps, and real gaps. To examine whether LR_Gapcloser supports the raw reads to fill the gaps and to compare the closure performance between using raw reads and corrected reads, in all tests the raw and corrected long reads were input to the tools.

#### Closure of gaps in the NGS-generated assemblies

We *de novo* assembled genomes with cleaned Illumina reads using Platanus [48] with the default parameters. Platanus is an efficient assembly approach using whole-genome shotgun short reads and has the best assembly performance compared to other existing assemblers. First, paired-end reads were assembled into contigs. Then, the contigs were scaffolded with paired-end and mate-paired reads. Finally, gap closure was conducted with paired-end reads. These NGS-derived assemblies were used in the downstream gap closure with the long reads.

For each species, we computed the six indicators for three gap-closed assemblies. Because the input NGS assembly contained misassemblies, the errors generated by the individual gap-closing tools were equal to the difference between all the observed errors in the gap-closed assembly and those in the input assembly.

#### Closure of repeat-derived gaps in the reference assemblies

Repetitive DNA is one of the most important factors that contribute to fragmented genome assemblies [49, 50]. To compare the ability of each tool to finish the gaps in the repeat regions, we generated repeat-derived gaps by replacing repeats with gaps of the same length. In the *S. cerevisiae* assembly, repeat regions longer than 200 bp were designated as gaps. In the *C. elegans* assembly, repeat regions longer than 1,000 bp were set as gaps. We generated 480 repeat-derived gaps, the maximal length of which was 20.2 kb. Before producing repeat-derived gaps in the HsX assembly, we removed the real gaps from the assembly to facilitate a comparison of the closure performances on repeat-derived gaps. A total of 1,044 repeat regions longer than 2,000 bp were then created as gaps. The maximal gap length was 20.6 kb.

The input scaffolds for gap closure were the same for the sequences of the corresponding reference genome and were thus error-free. By using the error-free contig sets, we can determine the number of misassemblies introduced by each tool as the observed errors in the gap-closed assembly.

#### Closure of real gaps in HsX of the CHM1 assembly and real gaps in the largest scaffold of the GM12878 assembly

The reference genomes of *S. cerevisiae* S288C and *C. elegans* Bristol were complete. However, the HsX sequence in CHM1 genome and the sequence of KQ079791 in GM12878 genome were unfinished. The HsX sequence contains 2,280 gaps with a contig N50 of 157.2 kb. With the PacBio reads, we performed a comparative analysis of the three tools on the closure of real gaps in the HsX assembly.

The largest sequence of KQ079791 from human GM12878 genome assembly contains 53 gaps with a contig N50 of 2.86 Mb. The performances of different tools using the Nanopore reads were compared by closing the real gaps in this scaffold.

### Using LR_Gapcloser to update the reference-quality assemblies by different approaches and from large and complex genomes

In order to assess the scalability and performance of LR_Gapcloser over a range of conditions, we applied LR_Gapcloser to assemblies generated by different approaches and originating from the large and complex genomes. In the above tests, we demonstrated that more gaps were closed using the raw long reads than using the corrected reads. Therefore, in this assessment we utilized the raw reads to close the gaps.

First, we examined the performance of LR_Gapcloser to improve the contiguity of three reference-quality human genome assemblies generated from different approaches. Steinberg et al. utilized end-sequenced BAC clones and 100 × Illumina sequencing reads with a reference guide strategy to generate a reference-quality assembly of the CHM1 genome [23]. The assembly had a total gap length of 210.2 Mb and the contig N50 size was as low as 143.9 kb. Pendleton et al. generated a hybrid assembly of GM12878 cell genome with a scaffold N50 value of 26.8 Mb [29]. The contig N50 size was 1.56 Mb and the total gap length was 146.4 Mb. Seo et al. generated a highly contiguous Korean genome assembly with a contig N50 size of 18.1 Mb and a scaffold N50 size of 44.8 Mb (NCBI AssemblyDB: GCA_001750385) [30]. The corresponding raw PacBio reads for these three assemblies were downloaded from the whole genome sequencing projects and used to close gaps.

Second, we assessed the scalability of LR_Gapcloser to improve the contiguity of genome assembly of a larger size and more complex genome than the human genome. The genome of *T. urartu*, the progenitor of wheat A subgenome, has an estimated size of 4.94 Gb and is characterized as repeat rich (81.4%). Ling et al. integrated BAC sequencing data, Illumina data, and PacBio reads and generated the *T. urartu* genome assembly, the size of which was 4.85 Gb. They used PBJelly and the raw PacBio reads to finish the gaps. The final gap length was 30 Mb. The contig N50 was 278 kb (NCBI Assembly accession: GCA_003073215.1). We utilized LR_Gapcloser and the raw long reads to further close the gaps in the assembly.

### Performance comparison of different hybrid assembly strategies by using TGS and NGS reads

In the genome sequencing projects in which both NGS and TGS reads were generated, although many strategies integrating the assembly, scaffolding, and gap closure were developed to generate assemblies, their performance and accuracy have not been investigated comprehensively. To provide an optimal hybrid strategy to generate a high-quality assembly, we evaluated the contiguity and correctness of six hybrid assembly strategies by combining TGS-based and NGS-based assemblers and LR_Gapcloser. Using the NGS and TGS reads in the above analysis as test datasets, six strategies were employed to generate the assemblies of *S. cerevisiae* S288C, *C. elegans* Bristol, and HsX. In strategy 1, Platanus [48] was employed to assemble cleaned Illumina reads from paired-end and mate-pair libraries, and the gaps were finished with paired-end libraries. In strategy 2, the gaps in the assemblies generated by strategy 1 were finally filled using LR_Gapcloser with the raw long reads. In strategy 3, PacBio reads were assembled using Canu [32], a scalable and accurate TGS-based assembler. In strategy 4, the assemblies generated by strategy 3 were further scaffolded using mate-pair libraries, and the gaps were closed with paired-end libraries using Platanus. In strategy 5, the gaps in the assemblies generated by strategy 4 were finally filled using LR_Gapcloser with the raw long reads. Unlike the above step-wise hybrid assemblies, strategy 6 used the hybridSPAdes algorithm [33] to assemble short and long reads simultaneously. We evaluated the contiguity and correctness of the assemblies generated by these six strategies compared with the reference genomes. Finally, based on the best performance strategy, we integrated the NGS and TGS reads to generate a new assembly of the human CHM1 genome.

## Availability of source code and requirements

Project name: LR_Gapcloser

Project home page: http://www.fishbrowser.org/software/LR_Gapcloser/

https://github.com/CAFS-bioinformatics/LR_Gapcloser

Operating system(s): linux

Programing language: perl, shell

Other requirements: none

License: GPLv3

RRID:SCR_016194

## Availability of supporting data

All supporting data including gap-closed assemblies are available at [51]. Snapshots of the code and further supporting data are available in the *GigaScience* repository, GigaDB [52].

## Additional files

**Figure S1:** Alignment of 18 BAC sequences to the new CHM1 genome assembly.

**Figure S2:** The runtime of tag fragmentation and alignment

**Table S1:** Whole genome Illumina sequencing reads obtained from NCBI SRA data

**Table S2:** The statistics of used Pacbio reads

**Table S3:** The statistics of used Nanopore reads

**Table S4:** The filled bases, runtime and memory usage of closing gaps in the NGS assemblies with Pacbio reads

**Table S6:** Gap-closure performance in the NGS-generated assemblies with Nanopore reads (for contigs ≥ 1000 bp)

**Table S7:** The filled bases, runtime and memory usage of closing gaps in the NGS assemblies with Nanopore reads

**Table S9:** The filled bases, runtime and memory usage of closing the repeat-derived gaps in the reference assemblies with Pacbio reads

**Table S11:** Closure on repeat-derived gaps in the reference assemblies with Nanopore reads (for contigs ≥ 1000 bp)

**Table S12:** The filled bases, runtime and memory usage of closing the repeat-derived gaps in the reference assemblies with Nanopore reads

**Table S14:** The filled bases, runtime and memory usage of closing the real gaps in HsX sequence with Pacbio reads

**Table S15:** The filled bases, runtime and memory usage of closing the real gaps in KQ079791 with Nanopore reads

**Table S17:** Summary statistics of different human *de novo* assemblies

**Table S18:** Sequence identities of 18 BAC sequences to the new CHM1 assembly

**Table S5:** Quast reports on gap-closure of NGS assemblies with Pacbio reads

**Table S8:** Quast reports on gap-closure of NGS assemblies with Nanopore reads

**Table S10:** Quast reports on closures of repeat-derived gaps with Pacbio reads

**Table S13:** Quast reports on closures of repeat-derived gaps with Nanopore reads

**Table S16:** Quast reports on assemblies of three species generate by different strategies

## Abbreviations

BAC: bacterial artificial chromosome; BWA-MEM: Burrows-Wheeler Aligner Maximal Exact Match; HsX: human chromosome X; NCBI, National Center for Biotechnology Information; NGS: next-generation sequencing; PacBio: Pacific Biosciences; SRA: Sequence Read Archive; TGS: third-generation sequencing.

## Consent for publication

Not applicable.

## Competing interests

The authors declare that they have no competing interests.

## Funding

This study was supported by National Natural Science Foundation of China (31672644) and the Special Scientific Research Funds for Central Non-profit Institutes, Chinese Academy of Fishery Sciences (2018HY-ZD0207 and 2018B004).

## Author contributions

We describe contributions for all authors to this paper using the CRediT taxonomy.

**Table:** 

Authors	Contributor Role
Gui-Cai Xu, Jiong-Tang Li	Methodology
Gui-Cai Xu	Software
Jiong-Tang Li, Tian-Jun Xu	Supervision
Gui-Cai Xu, Rui Zhu	Validation
Rui Zhu	Visualization
Yan Zhang, Shang-Qi Li	Resources
Jiong-Tang Li, Yan Zhang, Hong-Wei Wang	Funding Acquisition
Jiong-Tang Li	Conceptualization

## Supplementary Material

giga-d-18-00342_original_submission.pdf

giga-d-18-00342_revision_1.pdf

response_to_reviewer_comments_original_submission.pdf

reviewer_1_report_(original_submission) -- Fritz Sedlazeck 9/25/2018 Reviewed

reviewer_1_report_1_revision_1 -- Fritz Sedlazeck 11/12/2018 Reviewed

reviewer_2_report_(original_submission) -- Hamid Mohamadi, PhD 9/25/2018 Reviewed

Supplemental Files
