# Supplementary material for: LR_Gapcloser: a tiling path-based gap closer that uses long reads to complete genome assembly
Source: Gigascience. 2018 Dec 21;8(1):giy157. doi: 10.1093/gigascience/giy157 (PMC6324547; doi:10.1093/gigascience/giy157)
Supplement: giga-d-18-00342_original_submission.pdf [file giy157_giga-d-18-00342_original_submission.pdf]

GigaScience

LR_Gapcloser: a tiling path-based gap closer that uses long reads to complete genome assembly

--Manuscript Draft--

**Manuscript Number:**	GIGA-D-18-00342
**Full Title:**	LR_Gapcloser: a tiling path-based gap closer that uses long reads to complete genome assembly
**Article Type:**	Technical Note
**Funding Information:**	National Natural Science Foundation of China (31672644) — Dr Jiong-Tang Li<br>the Special Scientific Research Funds for Central Non-profit Institutes, Chinese Academy of Fishery Sciences (2018HY-ZD0207) — Dr Yan Zhang<br>the Special Scientific Research Funds for Central Non-profit Institutes, Chinese Academy of Fishery Sciences (2018B004) — Mr Hong-Wei Wang

National Natural Science Foundation of China (31672644)	Dr Jiong-Tang Li
the Special Scientific Research Funds for Central Non-profit Institutes, Chinese Academy of Fishery Sciences (2018HY-ZD0207)	Dr Yan Zhang
the Special Scientific Research Funds for Central Non-profit Institutes, Chinese Academy of Fishery Sciences (2018B004)	Mr Hong-Wei Wang

Abstract:

Background

Completing a genome is an important goal of genome assembly. However, many assemblies, including reference assemblies, are unfinished and have a number of gaps. Long reads obtained from third-generation sequencing (TGS) platforms can help close these gaps and improve assembly contiguity. However, current gap-closure approaches using long reads require extensive runtime and high memory usage. Thus, a fast and memory-efficient approach using long reads is needed to obtain complete genomes.

Findings

We developed LR_Gapcloser to rapidly and efficiently close the gaps in genome assembly. This tool utilizes long reads generated from TGS sequencing platforms. Tested on de novo assembled-gaps, repeat-derived gaps, and real gaps, LR_Gapcloser closed a higher number of gaps faster, with a lower error rate and a much lower memory usage than two existing, state-of-the art tools. This tool utilized raw reads to fill more gaps than when using error-corrected reads. It is applicable to gaps in the assemblies by different approaches and from large and complex genomes. After performing gap-closure using this tool, the contig N50 size of the human CHM1 genome was improved from 143 kb to 19 Mb, a 132-fold increase. We also closed the gaps in the Triticum urartu genome, a large genome rich in repeats, and the contig N50 size was increased by 40%. Further, we evaluated the contiguity and correctness of six hybrid assembly strategies by combining the optimal TGS-based and NGS-based assemblers with LR_Gapcloser. A proposed and optimal hybrid strategy generated a new human CHM1 genome assembly with marked contiguity. The contig N50 value was over 28 Mb, which is larger than previous non-reference assemblies of the diploid human genome.

Conclusions

LR_Gapcloser is a fast and efficient tool that can be used to close gaps and improve the contiguity of genome assemblies. A proposed hybrid assembly including this tool promises reference-grade assemblies. The software is available at http://www.fishbrowser.org/software/LR_Gapcloser/.

Corresponding Author: Jiong-Tang Li
Chinese Academy of Fishery Sciences
CHINA

Corresponding Author Secondary Information:

Powered by Editorial Manager® and ProduXion Manager® from Aries Systems Corporation

Corresponding Author's Institution:	Chinese Academy of Fishery Sciences
Corresponding Author's Secondary Institution:	
First Author:	Gui-Cai Xu
First Author Secondary Information:	
Order of Authors:	Gui-Cai Xu
	Tian-Jun Xu
	Rui Zhu
	Yan Zhang
	Shang-Qi Li
	Hong-Wei Wang
	Jiong-Tang Li
Order of Authors Secondary Information:	
Additional Information:	
Question	Response
Are you submitting this manuscript to a special series or article collection?	No
**Experimental design and statistics**<br><br>Full details of the experimental design and statistical methods used should be given in the Methods section, as detailed in our Minimum Standards Reporting Checklist. Information essential to interpreting the data presented should be made available in the figure legends.<br><br>Have you included all the information requested in your manuscript?	Yes
**Resources**<br><br>A description of all resources used, including antibodies, cell lines, animals and software tools, with enough information to allow them to be uniquely identified, should be included in the Methods section. Authors are strongly encouraged to cite Research Resource Identifiers (RRIDs) for antibodies, model organisms and tools, where possible.<br><br>Have you included the information	Yes

requested as detailed in our Minimum Standards Reporting Checklist?	
**Availability of data and materials**<br><br>All datasets and code on which the conclusions of the paper rely must be either included in your submission or deposited in publicly available repositories (where available and ethically appropriate), referencing such data using a unique identifier in the references and in the "Availability of Data and Materials" section of your manuscript.<br><br>Have you have met the above requirement as detailed in our Minimum Standards Reporting Checklist?	Yes

manuscript Click here to download Manuscript gigascience manuscript.doc

LR_Gapcloser: a tiling path-based gap closer that uses long reads to complete genome assembly

Gui-Cai Xu[1,2], Tian-Jun Xu[2], Rui Zhu[1,3] , Yan Zhang[1], Shang-Qi Li[1], Hong-Wei Wang[1], and Jiong-Tang Li[1,*]

[1] Key Laboratory of Aquatic Genomics, Ministry of Agriculture and Rural Affairs, CAFS Key Laboratory of Aquatic Genomics and Beijing Key Laboratory of Fishery Biotechnology, Chinese Academy of Fishery Sciences, Beijing, 100141, China

[2] College of Marine Science, Zhejiang Ocean University, Zhoushan, 316022, China

[3] College of Fisheries and Life Science, Shanghai Ocean University, Shanghai, 201306, China

* Correspondence to: Tel: +86 10 6867 3905; Fax: +86 10 6867 6685; Email: lijt@cafs.ac.cn

Abstract

Background

Completing a genome is an important goal of genome assembly. However, many assemblies, including reference assemblies, are unfinished and have a number of gaps. Long reads obtained from third-generation sequencing (TGS) platforms can help close these gaps and improve assembly contiguity. However, current gap-closure approaches using long reads require extensive runtime and high memory usage. Thus, a fast and memory-efficient approach using long reads is needed to obtain complete genomes.

Findings

We developed LR_Gapcloser to rapidly and efficiently close the gaps in genome assembly. This tool utilizes long reads generated from TGS sequencing platforms. Tested on *de novo* assembled-gaps, repeat-derived gaps, and real gaps, LR_Gapcloser closed a higher number of gaps faster, with a lower error rate and a much lower memory usage than two existing, state-of-the art tools. This tool utilized raw reads to fill more gaps than when using error-corrected reads. It is applicable to gaps in the assemblies by different approaches and from large and complex genomes. After performing gap-closure using this tool, the contig N50 size of the human CHM1 genome was improved from 143 kb to 19 Mb, a 132-fold increase. We also closed the gaps in the *Triticum urartu* genome, a large genome rich in repeats, and the contig N50 size was increased by 40%. Further, we evaluated the contiguity and correctness of six hybrid assembly strategies by combining the optimal TGS-based and NGS-based assemblers with LR_Gapcloser. A proposed and optimal hybrid strategy generated a new human CHM1 genome assembly with marked contiguity. The contig N50 value was over 28 Mb, which is larger than previous non-reference assemblies of the diploid human genome.

Conclusions

LR_Gapcloser is a fast and efficient tool that can be used to close gaps and improve the contiguity of genome assemblies. A proposed hybrid assembly including this tool promises reference-grade assemblies. The software is available at http://www.fishbrowser.org/software/LR_Gapcloser/.

Keywords: gap-closure, genome assembly, third-generation sequencing, next-generation sequencing, repetitive elements

Introduction

Next-generation sequencing (NGS) technologies allow for the low-cost and high-speed construction of genome sequences by *de novo* assembly. Along with the advantages of NGS technologies, over the last decade, many genome projects (for example, the 10K genome project [1] and 100K Pathogen Genome Project [2]) were initiated and the genomes of numerous species were assembled [3, 4]. However, factors like sequencing biases [5], repeat regions [6], and heterochromatin [7] make some regions difficult or impossible to assemble, leading to gaps and fragmented genome assemblies.

The gap-closure process is the last but most essential step for increasing the completeness and contiguity of genome assemblies. To obtain complete genomes, several gap-closure approaches including GapFiller [8], GapCloser [9], Sealer [10], GapBlaster [11], GapReduce [12], Gap2Seq [13], were developed to use NGS reads or pre-assembled contigs [14] to fill the gaps. However, these gap-closing tools display a high rate of misassembly during the gap-closing process [15]. Furthermore, it is hard to close all gaps, especially large ones, by using these tools. Long single-molecule sequencing technologies, also known as third-generation sequencing (TGS) technologies, for instance, Pacbio and Nanopore platforms, produce long and unbiased reads [16, 17], which have the potential to fill these gaps and achieve complete genome assemblies. PBJelly [18] and GMcloser [15] employ Pacbio reads for gap closure. PBJelly aligns long reads to the reference assemblies using BLASR [19], selects supporting reads, performs local gap assembly and decides upon an accurate assembly for gap filling. GMcloser splits scaffolds into sub-contigs, and aligns long reads to sub-contigs using MUMmer [20] or blastn. GMcloser uses likelihood-based classifiers to correctly assign long reads to gaps in scaffolds. However, their disadvantages, including long runtime, high memory usage and low closure performance, limit their application, especially into large and complex genomes. Therefore, a fast and memory-efficient gap closure approach is required to fill gaps in genome assemblies.

Herein, we developed LR_Gapcloser to efficiently and rapidly close gaps in assemblies using long reads. Many notable advantages were exhibited compared to previous gap-closing tools; these include higher gap closure performance, less runtime, fewer peak memories, and lower misassemblies. Even for both large and complex genomes and repeat-derived gaps, this tool exhibited better performance. Finally, for the genomes that were sequenced using both NGS and TGS technologies, we evaluated the contiguity and correctness of different hybrid assembly strategies

and proposed an optimal hybrid strategy combining TGS-based and NGS-based assemblers with LR_Gapcloser to produce high-quality assemblies.

Findings

The algorithm of LR_Gapcloser

The primary steps of the LR_Gapcloser algorithm are outlined in Figure 1. This tool can use either uncorrected or error-corrected long reads to fill gaps.

(1) Fragmentation of long reads to short tags in a tiling path

Each long read was fragmented into short tags of the same length (default length: 300 bp). All the tags were distributed in an orderly fashion along one long read without overlapping. The distribution of all the tags formed a tiling path along this read.

(2) Alignment and filtration

All the tags were aligned to genome sequences using the BWA-MEM algorithm [21]. We calculated the coverage of each alignment based on the following formula:

$$\text{Coverage} = \frac{(match\ base\ length + insert\ base\ length)}{tag\ length}$$

We adopted the definitions of the match base and the insert base as described in the SAM format specification [22]. The match base length is the sum of identical bases between one read and its aligned sequence. The insert base length is the total number of bases inserted into the reference genome sequence. If the coverages of the alignments were over a certain threshold (default coverage of 80%), then these alignments were retained. We classified the tags into the following two types: (1) tags uniquely aligned to one genomic locus, and (2) tags having at least two aligned positions. We performed three rounds of filtrations.

For the tags of type 1, we employed the following strategies to refine the orientations and orders of uniquely aligned tags. First, the orientations of uniquely aligned tags in one scaffold possibly conflicted with one another due to the sequencing error in the long read or the misassemblies in this scaffold. Based on the alignments of the tags of type 1, we calculated the tag numbers of each orientation. If the tag number from each alignment orientation was equal, then all the alignments from these reads to this scaffold were potentially error-prone and thus removed. If one alignment orientation had more uniquely aligned tags than the other orientation, then all alignments of the former

orientation were retained and alignments of the latter orientation were removed. Second, the order of uniquely aligned tags in one read might not be consistent with the order in the corresponding scaffold due to misalignment or misassembly. If a read had only two retained tags and the order of these two tags in one read were consistent with the order in the scaffold, these tags were retained. Otherwise, these tags were removed. If a read had more than two retained tags, then we selected the tag at the median position on the read and its position on the scaffold as reference. We compared the positions of the other tags with the position of the reference tag. If the order of the compared tag and the reference tag on the read were consistent with the order on the scaffold, then this compared tag and its alignment were retained; otherwise, it was removed.

After we refined the alignment of the tags of type 1, we searched the best alignment for each tag of type 2 using the following two criteria: (i) the aligned scaffold and corresponding orientation should be the same as that of the neighbouring tags of type 1; and (ii) the relative position of this tag compared to the neighbouring tags of type 1 on the read should be consistent with the one on the scaffold.

(3) Selection of supporting reads and gap closure

We tallied the gaps into the following three types: (i) two boundaries of one gap had aligned tags from one read. The tag number at each boundary should be over a threshold (default number of 5) to ensure accurate alignments. Tags at one boundary formed pairs with tags at the other boundary. Assuming that two tags, ***a*** and ***b***, from read (**A**) were aligned to two boundaries of a gap (**B**), then these two tags formed a pair. We retained this pair in the downstream analysis if it satisfied the following condition:

$$1-\boldsymbol{c} < \frac{p(\boldsymbol{b})-p(\boldsymbol{a})}{O(\boldsymbol{b})-O(\boldsymbol{a})} < 1+\boldsymbol{c}$$

where p(***a***) and p(***b***) were the positions in read (**A**), O(***a***) and O(***b***) were the positions in the aligned scaffold, and the variable ***c*** was the allowed length derivation between the distance in the read and the one in the scaffold (default setting as 0.2).

The retained tag pair was then used as supporting evidence for this read to close the gap. For each gap, the read with the most tag pairs was selected and the sequence between the two tags nearest to the two boundaries of the gap was used to fill the gap.

(ii) Two boundaries of one gap had multiple tag alignments from different reads. At each boundary, if the read had more than a minimum number (default number of 5) of aligned tags and it covered the

largest proportion of the gap, this read was selected. Two reads were retained to fill the gap. For each read, we determined the tag nearest to the gap and the sequence from this tag to the end of the read was used to fill the gap.

We assumed that a tag (***a***) from read (**A**) and another tag (***b***) from read (**B**) were aligned nearest to each boundary of gap (**C**). The lengths of read (**A**), read (**B**) and gap (**C**) were L(**A**), L(**B**), and L(**C**), respectively. The position of tag (***a***) in read (**A**) was p(***a***) and the position of tag (***b***) in read (**B**) was p(***b***). If the sum of the bases closed by these two reads was smaller than 100%, that is,

$$\mathrm{L}(\boldsymbol{A}) - \mathrm{p}(\boldsymbol{a}) + \mathrm{p}(\boldsymbol{b}) < L(\boldsymbol{C}),$$

then this gap was not completely closed. A sequence composed of the letter 'N' was inserted between two filled sequences. The number of inserted 'N's was the difference between the gap length and the length of the filled sequence, where

$$\mathrm{N} = \mathrm{L}(\boldsymbol{C}) - \left(L(\boldsymbol{A}) - p(\boldsymbol{a}) + p(\boldsymbol{b})\right).$$

If the sum of bases closed by these two reads was larger than or equal to 100%, that is,

$$\mathrm{L}(\boldsymbol{A}) - p(\boldsymbol{a}) + p(\boldsymbol{b}) \geq L(\boldsymbol{C}),$$

then the sequence from p(***a***) to the end of read (**A**) was retrieved. The sequence from the start of read (**B**) to p(***b***) was retrieved and connected to the above sequence to fill the gap. One hundred 'N's were inserted between two filled sequences to indicate a gap that could be possibly completed.

(iii) Only one boundary of one gap had multiple tag alignments from different reads. The read covering the largest proportion of the gap was selected. After the tag nearest to the gap was determined, the sequence from it to the end of the read was used to fill the gap. We inserted a sequence composed of the letter 'N'. The number of inserted 'N's was the difference between the gap length and the length of the filled sequence.

The entire gap-closing process can be iteratively implemented. Gaps in the types described in (ii) and (iii) would be further completed as the type described in (i) following the subsequent next iteration of gap closure.

Closure of gaps in NGS-generated assemblies

All public sequencing reads used in this study are described in Additional file 1: Table S1, S2 and S3 (see 'Sequencing read sets and selected reference genomes' below). We first examined the performance of each tool (LR_Gapcloser, PBJelly [18] and GMcloser [15]) on NGS assemblies of *S. cerevisiae* S288C, *C. elegans* Bristol, and human CHM1 chromosome X (HsX hereafter) using the

raw Pacbio reads. The raw reads were not corrected and were directly fed into these three tools. For *S. cerevisiae*, which has a small genome, the contig N50 of the NGS assembly was 43.4 kb (Table 1). It included 421 gaps with a total length of 290.8 kb and a maximal gap length of 13.5 kb (Additional file 1: Table S4). Compared against the reference assembly, the input assembly included 29 misassemblies and covered 5,958 complete genes. LR_Gapcloser filled the most bases (99.97%; Additional file 1: Table S4, and Additional file 2: Table S5) at the lowest error rate (the ratio of misassemblies to contig N50: 3.8×10^{-5}, Table 1). LR_Gapcloser led to the greatest increase in the contig N50 (18.1 fold) and had a runtime of 24 minutes, approximately 1.6% of the runtime of GMcloser (the second-fastest tool using 24 hours 30 minutes, Additional file 1: Table S4). The peak memory usage of LR_Gapcloser was only 3.8 % of that by GMcloser and 18.6% of that by PBJelly.

For *C. elegans*, a medium-sized genome, the contig N50 of the NGS assembly was 43.2 kb. This assembly had 4,452 gaps, which accounted for 3.67 Mb, with a maximal length of 12.5 kb. It included 73 misassemblies and covered 42,116 complete genes (Table 1). LR_Gapcloser also exhibited the best performance among the three tools. After gap-closure by LR_Gapcloser, at the lowest error rate (the ratio of misassemblies to contig N50: 15×10^{-5}), the most gap bases (99.7%) were filled, leading to the greatest increase in the contig N50 (60.3 fold) and the greatest decrease in the contig number (Table 1, Additional file 1: Table S4 and Additional file 2: Table S5). The assembly closed by LR_Gapcloser covered the highest number of complete genes (43,841). Furthermore, LR_Gapcloser had the shortest runtime (4 hours and 25 minutes), 7.5% of the runtime of GMcloser (the second-fastest tool, running over 59 hours, Additional file 1: Table S4). In addition, the peak memory usage of LR_Gapcloser was much lower than that of the other two tools.

A similar tendency was observed for the HsX assembly. The contig N50 of the NGS assembly was 76.5 kb. It included 3,737 gaps with a total length of 2.05 Mb. LR_Gapcloser filled the most nucleotides (98.8%) to gaps at the lowest error rate (the ratio of misassemblies to contig N50: 18×10^{-5}, Table 1, Additional file 1: Table S4 and Additional file 2: Table S5). This tool produced the greatest increase of the contig N50 (34.1 fold). The runtime of LR_Gapcloser was the shortest (3 hours and 53 minutes), at approximately 32.6% of the runtime of PBJelly (the second fastest tool with a runtime of approximately 12 hours, Additional file 1: Table S4). The comparison on the NGS-produced gaps demonstrated that the efficiency, accuracy, and speed of LR_Gapcloser were

considerably higher than those of the other tools. These results also suggested that for assemblies of small contigs, the contig N50 size could be significantly improved.

We then tested the performance of each tool on these three NGS assemblies using the error-corrected Pacbio reads, and demonstrated that among the three tools, LR_Gapcloser closed the most gaps at the lowest error rate. LR_Gapcloser required a shorter runtime and lower peak memory than the other two tools (Table 1, Additional file 1: Table S4). A similar fast and efficient performance of LR_Gapcloser was also observed when closing gaps in the NGS-generated assemblies of *S. cerevisiae* and *C. elegans* using either the raw Nanopore reads or error-corrected Nanopore reads (Additional file 1: Table S6 and Table S7, Additional file 3: Table S8).

Closure of repeat-derived gaps

Due to their high similarity, repetitive regions are difficult to close, creating a challenge to complete genome assembly. We evaluated the closure performances of the above three tools on the repeat-derived gaps using the raw Pacbio reads, the error-corrected Pacbio reads, the raw Nanopore reads, and the error-corrected Nanopore reads. We first tested the performance of each tool using the raw Pacbio reads. The raw reads were not corrected and directly used as input for the three tools. In the *S. cerevisiae* assembly, we designated 464 repeat-derived gaps, the total size of which was 513.4 kb (Table 2, Additional file 1: Table S9). The gap sizes ranged from 200 bp to 11,510 bp. These gaps broke the reference genome into an error-free contig set including 299 contigs, in which the contig N50 size was 77.84 kb. The new assembly generated by GMcloser was 20.7 Mb, much larger than the reference genome size (12.1 Mb), indicative mis-closures caused by GMcloser (Additional file 4: Table S10). The runtime of LR_Gapcloser was 44 minutes, only 2.9% of PBJelly runtime (Additional file 1: Table S9). The peak memory usage of LR_Gapcloser was also the lowest, 13.5% of the usage required by PBJelly. LR_Gapcloser filled the most gap bases with 99.4% at the lowest error rate (the ratio of misassemblies to contig N50: 0.98×10^{-5}, Additional file 1: Table S9). This tool led to the greatest increase in the contig N50 (10.4 fold) with the highest number of complete genes (6,403) (Additional file 4: Table S10).

In the *C. elegans* assembly, 480 repeat-derived gaps were created with a total size of 964.7 kb (Additional file 1: Table S9). The error-free set included 406 contigs longer than 1 kb. The contig N50 size was 510.8 kb (Table 2). In the new assembly by LR_Gapcloser, in total 92.5% of gap bases were filled (Additional file 1: Table S9). The contig N50 was increased to 10.4 Mb with a 20.3-fold

improvement. The complete gene number was the highest (44,794, Additional file 4: Table S10) and the ratio of misassemblies to contig N50 was the lowest (0.35×10^{-5}). LR_Gapcloser was also faster and had lower peak memory usage than the other two tools (Additional file 1: Table S9).

We produced 1,044 repeat-derived gaps in the HsX assembly, the total size of which was 3.43 Mb. The error-free set consisted of 977 contigs over 1 kb. The similar fastest and best performance of LR_Gapcloser was observed in this comparison to the other two tools. Our tool added the most nucleotides to the gap regions (99.6%) at the lowest error rate (the ratio of misassemblies to contig N50: 0.31×10^{-5}) and had the shortest runtime of 5 hours and 33 minutes (Additional file 1: Table S9). The contig N50 size increased from 289.2 kb to 16 Mb (a 55.4-fold increase). The gap-closed assembly covered the highest proportion of the reference genome (99.95%, Table 2).

Comparing the performances of three tools on these repeat-derived gaps using the error-corrected Pacbio reads, the raw Nanopore reads, and the error-corrected Nanopore reads, further demonstrated that among the three tools, LR_Gapcloser closed the most gaps at the lowest error rate with the shortest runtime and lowest peak memory (Table 2, Additional file 1: Table S11, Table S12, and Additional file 2: Table S13). These results also support that our tool could be an efficient solution for closing gaps in repetitive regions.

Closure of real gaps

We further tested the closure performances of the three tools on real gaps in the HsX assembly using either the raw Pacbio reads or error-corrected reads. Although 2,279 contigs were anchored to the HsX sequence (155 Mb) using a reference-guide assembly strategy [23], the contig N50 size was only 157 kb. The longest gap located at the centromere was 3 Mb. The total size of the other 2,278 gaps accounted for 2.49 Mb. When using the raw Pacbio reads, although LR_Gapcloser had a longer runtime than PBJelly, LR_Gapcloser made the greatest increase of contig N50 from 157 kb to 4.07 Mb and decreased the contig number from 2,280 to 164 (Additional file 1: Table S14). When using the error-corrected Pacbio reads, we observed the same optimal performance of LR_Gapcloser.

To test the scalability and performance of the tools in closing real gaps with Nanopore data, we closed the gaps in the largest scaffold (KQ079791) of human GM12878 assembly using these three tools. This scaffold had 53 gaps and the contig N50 size was 2.86 Mb. We found that when using either the raw Nanopore reads or error-corrected Nanopore reads, LR_Gapcloser closed the most gaps, had the shortest runtime, and required the lowest peak memory (Additional file 1: Table S15).

Improving the contiguity of the reference-quality assemblies by different approaches and from large and complex genomes

Overall, the above three comparisons on NGS-generated gaps, repeat-derived gaps and real gaps, revealed that LR_Gapcloser closed more gaps with remarkably smaller error rates than the other methods. In addition to mate pair-based scaffolding [24], which was commonly used in genome projects, other assembly strategies have been used for genome assembly, and they can produce reference-quality assemblies with much longer contigs. These strategies include reference-guide assembly [25], the third generation of the single-molecule sequencing strategy [26], and next-generation mapping [27]. These strategies generated reference-quality assemblies with a much higher scaffold N50 size, although there were still gaps. Thus, we assessed the performance of LR_Gapcloser in improving the contiguity of the reference-quality genome assemblies by a range of approaches.

The reference CHM1 genome assembly included 40,893 contigs, which were anchored to 23 chromosomes with a reference guide strategy [23]. The assembly included 40,915 gaps (a total length of 210.2 Mb). The contig N50 size was 143.9 kb with the longest contig size of 7.16 Mb (Table 3). Using the raw Pacbio reads, LR_Gapcloser incorporated novel nucleotides of 30.2 Mb into the assembly. The number of gaps and contigs decreased to 522 and 508, respectively. Notably, the contig N50 size increased to 19.08 Mb and the largest contig increased to 57.1 Mb. The updated contig N50 size was larger than that generated by MHAP (4.32 Mb) [26] and MECAT (4.88 Mb) [28], both of which are TGS-based *de novo* assemblers.

The GM12878 cell genome assembly was generated by combining single-molecule sequencing reads with single-molecule genome maps [29]. Although the scaffold N50 value was over 26.8 Mb, the contig N50 was 1.56 Mb and the total gap size was 146.4 Mb. Using the raw Pacbio reads, LR_Gapcloser incorporated novel nucleotides of 95.7 Mb into the assembly. The gap number was reduced by 91%. Remarkably, the contig N50 size increased from 1.56 Mb to 12.99 Mb, and the largest contig was extended from 10.9 Mb to 49.2 Mb (Table 3).

PacBio sequencing, BioNano mapping, linked reads, and bacterial artificial chromosome (BAC) sequencing approaches were integrated to produce a high-quality Korean human genome assembly [30]. The high contiguity was represented by a contig N50 size of 18.1 Mb and a total gap size of 37.3 Mb. Our tool added 21.4 Mb (57.3%) of novel nucleotides to this assembly. The contig N50 size

increased to 23.4 Mb (Table 3) and the longest contig was extended to 85.2 Mb. These applications suggested that LR_Gapcloser was suitable for the contiguity improvement of genome assemblies generated by different strategies, even in high-quality assemblies.

We demonstrated the efficient performance of LR_Gapcloser when applied to large genomes as humans. The scalability of this tool to close gaps in larger genome assemblies was further examined by using it to close the gaps in the *Triticum urartu* genome, the progenitor of wheat A subgenome [31]. This genome is 4.94 Gb in size and repeat-rich. Although some gaps were closed in this genome by using PBJelly, there was still a total gap size of 30 Mb. The contig N50 size and longest contig size were 278.4 Kb and 2.07 Mb, respectively. Our tool added 11 Mb (36.7%) of novel nucleotides to the gap regions. The contig N50 size and longest contig size increased to 389.3 Kb and 3.67 Mb, respectively. These data demonstrated that this method is applicable on the small-, medium-, and large-sized genomic spectrum.

A proposed hybrid assembly strategy using TGS and NGS reads

The TGS-based assembly exhibits high contiguity, represented by a large contig N50 value [32]. The advantage of NGS-based assembly is represented by a large scaffold N50 value. For species in which both NGS and TGS reads were generated, we posed a question how to integrate the assembly, scaffolding, and gap-closure to generate a reference-grade genome assembly. There are currently six assembly strategies that have been developed to combine the NGS and TGS reads (see 'Performance comparison of different hybrid assembly strategies by using TGS and NGS reads' below). We applied these strategies to assembling the genomes of *S. cerevisiae*, *C. elegans*, and HsX, and evaluated the contiguity and correctness of each strategy.

In *S. cerevisiae*, the assemblies produced by the strategies with the TGS based-assembler (Strategy 3, 4, and 5) had higher genome coverages, higher contiguity and lower error rates than those produced with the NGS based-assembler (Strategy 1 and 2) and hybridSPAdes [33] (Strategy 6). Although the misassembly number and the ratio of misassemblies to contig N50 (NGA50) in the assembly by Strategy 5 ranked third, which were slightly lower than those by Strategy 3 and 4, all the other metrics including the coverage and contiguity of this assembly ranked first. The genome coverage of this assembly reached 99.5%. The sequence identity compared to the reference genome was as low as 5.23 mismatches and 18.78 indels per 100 kb (corresponding to 99.97% accuracy) (Additional file 4: Table S16). The new assembly generated by Strategy 5 had a scaffold N50 value of

834.2 kb and a contig N50 value of 834.2 kb (Table 4). The assembly completed 14 out of 17 chromosomes (Figure 2A). The other three chromosomes were spanned by just two scaffolds. These results reveal the perfect continuity of the assembly under Strategy 5.

In *C. elegans*, we found that the assemblies solely relying on either TGS reads or NGS reads (Strategy 1 and 3) had lower contig N50 sizes than the assemblies using the step-wise hybrid assembly of TGS and NGS reads (Strategy 2, 4 and 5). Apart from the ratio of misassemblies to NGA50, all other metrics of this assembly by Strategy 5 ranked first. This assembly consisted of 83 scaffolds and 83 contigs without any gaps and had the highest scaffold N50 value (12.89 Mb) and contig N50 value (12.89 Mb, Table 4). The genome coverage reached 99.26%. The sequence identity compared to the reference genome was higher than 99.93% (14.26 mismatches and 56.72 indels per 100 kb) (Additional file 4: Table S16). The longest scaffold approached 18.67 Mb, covering 89.1% of chromosome V. Five autosomal chromosomes (V, IV, II, I, and III) were largely spanned by two scaffolds (Figure 2B). The high coverage and contiguity with the low error rate demonstrated that Strategy 5, along with a sufficient coverage of long reads and short paired-end reads, could generate *de novo*-assembled genomes that approach reference quality.

We also observed the best performance of Strategy 5 in the HsX assembly. This assembly had the best value for all the metrics, except for the genome coverage (98.75%). Despite the original CHM1 Genome Project, including deep-coverage Illumina reads and BAC clones, the contig N50 of the new HsX assembly was greater than that of the reference genome size (7.87 Mb *vs* 157 kb) with fewer contigs (156 vs 2,279). Compared with the reference HsX assembly, the new assembly had an accuracy of 99.83% (13.22 mismatches and 156.98 indels per 100 kb) (Additional file 4: Table S16). Furthermore, the new HsX assembly was structurally consistent with the reference assembly (Figure 2C).

Strategy 5, which consisted of using TGS-based and NGS-based assemblers and LR_Gapcloser, had the best performance and the lowest error rate to generate reference-grade assemblies. We applied this strategy into generating a new, highly contiguous CHM1 assembly. The new assembly consisted of 2,265 scaffolds with a scaffold N50 size of 28.45 Mb (Additional file 1: Table S17). It achieved marked contiguity with the largest contig N50 value (28.45 Mb) thus far (except the GRCh38 reference assembly). This value is an order of magnitude larger than the CHM1 reference assembly generated by the earlier method (143.9 kb) [23], and it is even larger than previous non-reference

assemblies of the human diploid genome using long-range physical mapping strategies [29, 30, 34, 35] (Additional file 1: Table S17). To validate the assembly, according to the approach by Chaisson *et al.* [36], we compared 18 finished CHM1tert BAC clones [26, 36, 37] to the new assembly with MUMmer [20]. The assembly was structurally consistent with these BACs (Additional file 1: Figure S1). Single-nucleotide polymorphisms (SNPs) identified with 'show-snps' in the 'dnadiff' [38] package revealed 99.96% shared identity (Additional file 1: Table S18). Comparing our assembly to the GRCh38 assembly showed a general agreement between both assemblies (Figure 2D). These comparisons and validations suggest that the optimal and step-wise hybrid assembly strategy can generate the most contiguous genome assemblies for species in which both NGS and TGS reads were generated.

Discussion

Compared with the existing two tools, here we summarize the advantages exhibited by our tool, LR_Gapcloser. First, our tool is fast and efficient with low memory-usage. We designed three types of tests to compare the performances of the three tools. In all tests, LR_Gapcloser added the most nucleotides and closed the most gaps. In case of the ability to close NGS-generated gaps and repeat-derived gaps, LR_Gapcloser was 1.18~52.25 fold faster than PBJelly and 1.57~76.98 fold faster than GMcloser. In addition, the memory-usage of LR_Gapcloser was the lowest, requiring only 3.2%~28.0% of the peak memory usage by GMcloser and 8.4%~32.9% of that by PBJelly. Second, LR_Gapcloser efficiently uses the raw long reads to close the gaps. When using the raw long reads, GMcloser fill few gaps, while LR_Gapcloser and PBJelly could utilize the raw reads to finish the gaps. However, LR_Gapcloser added more nucleotides than PBJelly. Third, LR_Gapcloser produced the fewest misassemblies. As shown in Table 1 and 2, both the ratio of misassemblies to contig N50 and the ratio of misassemblies to contig NGA50 by LR_Gapcloser were much lower than that by the other two tools. Fourth, LR_Gapcloser has the best performance when applied to repeat-rich genomes. Repeats produce the greatest challenge to genome assembly. Tested on the repeat-derived gaps in the assemblies of different species, LR_Gapcloser closed at least 85.4% of the gaps, more than PBJelly and GMcloser. RepeatMasker [39] annotations of the added nucleotides to the real gaps in HsX by LR_Gapcloser indicate that over 52% of the closed regions originate from repeats. Fifth, the fast and memory-efficient characteristics of LR_Gapcloser suggest that the present method is suitable

for closing gaps in large assemblies. With 30 threads, PBJelly took 119 hours (Additional file 1: Table S4) with the uncorrected Pacbio reads to finish the gaps in NGS-generated repeats in *C. elegans* (genome size of 0.1 Gb and gap size of 3.6 Mb). However, with 30 threads LR_Gapcloser only required 63 hours to close the gaps in the *Triticum urartu* genome (genome size of 4.94 Gb and gap size of 30 Mb).

To achieve a rapid and optimal performance, we integrated three efficient approaches within LR_Gapcloser. First, it performs a strategy involving long read fragmentation and alignment. This strategy is 1.74~8.06-fold faster than the gap-closure tools that align the whole reads (Additional file 1: Figure S2). Second, we constructed a pipeline to select the best aligned reads for gap-closure. PBJelly selects reads overlaying the gaps and perform consensus calling to finish the gaps. Without consensus base calling or assembly in the gap regions, the selection strategy used in LR_Gapcloser was much faster and generated fewer misassemblies than the consensus calling strategy. Third, many processes in LR_Gapcloser were parallelized in the gap closing pipeline, including tag alignment to scaffolds, alignment coverage calculation, and filtration.

Over the last decade, the genomes of thousands of species have been sequenced using NGS technologies. The assemblies by NGS-based assemblers had large N50 sizes but small contig N50 sizes (Table 4). Long reads generated by TGS technologies provides promise for reconstructing complex genomic regions, including the repeat-rich heterochromatic regions of eukaryotic chromosomes. The hybrid and step-wise assembly of Strategy 5 using the TGS and NGS reads could produce assemblies having both a high scaffold N50 value and a high contig N50 value (Table 4). In the best case, an entire chromosome was assembled into a single sequence from telomere to centromere. Our results showed that 14 entire *S. cerevisiae* chromosomes were resolved into single sequences. In addition to the high contiguity, this optimal strategy generates assemblies with comparable accuracy (for example, 99.83% accuracy in a new HsX assembly) to those by NGS-based strategies (Strategy 1: 99.99% accuracy) (Additional file 4: Table S16). To improve the base quality of the assemblies generated by this optimal strategy, the assemblies can be further polished with NGS data.

LR_Gapcloser is flexible in that both Pacbio reads and Nanopore reads [40] can be utilized to fill the gaps. This tool is also likely to use the 10X genomic linked reads [41] and pre-assembled contigs to fill gaps in the assemblies. One limitation to the performance of LR_Gapcloser is that the current

read length could not efficiently complete the extremely long gaps, especially in the centromeric and telomeric regions. Therefore, one future improvement is to generate new strategies to produce longer reads.

Methods

Sequencing read sets and selected reference genomes

We selected reads and reference genomes by implementing the following strict criteria: (1) the genome is complete or almost finished to facilitate the measurement of the gap-closure accuracy. (2) Pacbio long reads (or Nanopore long reads), NGS reads, and the reference genome assembly should be generated from the same strain to avoid misassemblies resulting from sequence variations between strains. The reference genomes of three species *(S. cerevisiae* S288C, *C. elegans* Bristol, and *H. sapiens* CHM1) are characterized by their different genome sizes and levels of complexity, and were tested in this study. The reference genome assemblies and corresponding annotations of genes and repeats were taken from AsssemblyDB [42].

The raw Illumina sequencing reads were downloaded from the NCBI SRA database (Additional file 1: Table S1). The reads were then trimmed using SolexaQA [43]. Pairs in which both high-quality ends were longer than 25 bp were retained. We also created random Illumina mate-paired reads with large insert sizes from the reference genome using DWGSIM [44] with the default parameters (Additional file 1: Table S1). For *S. cerevisiae*, one real paired-end library (insert size of 300 bp), one real mate-end library (insert size of 3,400 bp), and three simulated mate-end libraries (insert sizes of 5,000, 10,000 and 15,000 bp) with 570 x genome coverage were input into the assembly. For *C. elegans*, this dataset included a real paired-end library (insert size of 250 bp) and four simulated mate-pair libraries (insert sizes of 3,000, 5,000, 10,000 and 15,000 bp). The sequencing coverage was over 380 x. For *H. sapiens*, the dataset included one real paired-end library (insert size of 300 bp), two real mate-pair libraries (insert sizes of 3,000 and 8,000 bp) and two simulated mate-pair libraries (insert sizes of 10,000 and 15,000 bp).

The sources of all long reads used in this study are listed in Additional file 1: Table S2 and Table S3. The raw reads (Pacbio and Nanopore) for *S. cerevisiae* and *C. elegans* were obtained from the NCBI SRA database and other websites, respectively. MECAT [28] corrected the raw reads. Both the raw and error-corrected reads were employed into the comparison of the gap-closure performances.

For a gap-closure comparison of the human CHM1 genome with Pacbio reads, we used only chromosome X (HsX) as a representative for the whole genome. We selected this small chromosome, 1/20 the size of the genome (155 Mb), because the existing gap-closers would take many days and require high memory usage to finish the gaps of the CHM1 genome (NCBI AssemblyDB: GCA_000306695). The Illumina reads were mapped against the entire genome using the BWA-MEM algorithm in the BWA package [45]. If at least one read on one Illumina pair was mapped to HsX, then this pair was employed into the *de novo* assembly of HsX. We also mapped the raw Pacbio reads to the reference genome using BWA-MEM (with the parameter of –x pacbio) and extracted the long reads mapped to HsX. The mapped raw reads were corrected using MECAT. Both the raw and error-corrected Pacbio reads were inputted into the comparison of the closure performances on the NGS-generated gaps, repeat-derived gaps, and real gaps.

Further, since there is no available Nanopore reads from human CHM1 genome, to compare the closure performances of different tools on real gaps with Nanopore reads, the Nanopore reads from human GM12878 genome were used (NCBI AssemblyDB: GCA_001013985) [46]. Following the above strategy, we selected the large scaffold (NCBI Accession: KQ079791) to represent the whole genome, with a length of 80.3 Mb. All raw Nanopore reads were firstly mapped against the GM12878 genome assembly using the BWA-MEM algorithm (with the parameter of -x ont2d). The raw reads mapped to this scaffold were then corrected using MECAT. The raw and error-corrected Nanopore reads were used to close gaps.

Evaluation of gap closure performance by three tools

We compared the gap closure performance of LR_Gapcloser and two currently available tools, GMcloser [15] and PBJelly [18]. These two tools were run using the default parameters. We ran each tool with 30 threads on the same machine. We estimated the performance of each tool using following six indicators: (1) closed gap number, (2) filled base number, (3) runtime, (4) peak memory usage, (5) quality metrics by Quast [47], and (6) misassembly ratio.

For each tool, the closed gap number was the difference between the gap numbers in the two assemblies before and after gap closure. The filled base number was the difference between the total gap length in the closed assembly and the one in the input assembly. To evaluate the gap closure accuracy, each assembly was split into contigs, which were compared against the reference genome using Quast [47] with the parameters of --min-alignment 500 and --min-identity 90. To evaluate the

quality of the genome assembly, Quast introduces a series of quality indicators, which are grouped into four categories: (i) contig sizes, (ii) misassemblies and structural variations, (iii) genome representation and its functional elements, and (iv) variations in N50 based on aligned blocks. Quast defines one misassembly in one contig as a position where: (i) the left flanking sequence and the right flanking sequence are over 1 kb apart on the same reference sequence, or (ii) these two flanking sequences have an overlapping region over 1 kb, or (iii) they align on opposite strands of the same genomic sequence or two different chromosomes [47].

It is reasonable that more misassemblies were generated as more gaps were finished and the contig N50 size increased, thus we introduced another two ratios to normalize the misassemblies:

- *Ratio of misassemblies to contig N50*: The total number of misassemblies divided by the contig N50 size.
- *Ratio of misassemblies to NGA50*: The total number of misassemblies divided by the NGA50 size. The contigs in the new assemblies are split into aligned blocks and the NGA50 is the NG50 of the aligned blocks.

Comparison of the performances of three tools on gaps of three types from different species

We compared the gap-closure performances of the three tools on three types of gaps: NGS-generated gaps, repeat-derived gaps, and real gaps. To examine whether LR_Gapcloser supports the raw reads to fill the gaps and to compare the closure performance between using raw reads and corrected reads, in all tests the raw and corrected long reads were inputted to the tools.

(1) Closure of gaps in the NGS-generated assemblies

We *de novo* assembled genomes with cleaned Illumina reads using Platanus [48] with the default parameters. Platanus is an efficient assembly approach using whole-genome shotgun short reads and has the best assembly performance compared to other existing assemblers. First, paired-end reads were assembled into contigs. Then, the contigs were scaffolded with paired-end and mate-paired reads. Finally, gap closure was conducted with paired-end reads. These NGS-derived assemblies were used in the downstream gap closure with the long reads.

For each species, we computed the six indicators for three gap-closed assemblies. Because the input NGS assembly contained misassemblies, the errors generated by the individual gap-closing tools were equal to the difference between all the observed errors in the gap-closed assembly and those in the input assembly.

(2) Closure of repeat-derived gaps in the reference assemblies

Repetitive DNA is one of the most important factors that contribute to fragmented genome assemblies [49, 50]. To compare the ability of each tool to finish the gaps in the repeat regions, we generated repeat-derived gaps by replacing repeats with gaps of the same length. In the *S. cerevisiae* assembly, repeat regions longer than 200 bp were designated as gaps. In the *C. elegans* assembly, repeat regions longer than 1,000 bp were set as gaps. We generated 480 repeat-derived gaps, the maximal length of which was 20.2 kb. Before producing repeat-derived gaps in the HsX assembly, we removed the real gaps from the assembly to facilitate a comparison of the closure performances on repeat-derived gaps. A total of 1,044 repeat regions longer than 2,000 bp were then created as gaps. The maximal gap length was 20.6 kb.

The input scaffolds for gap closure were the same for the sequences of the corresponding reference genome and were thus error-free. By using the error-free contig sets, we can determine the number of misassemblies introduced by each tool as the observed errors in the gap-closed assembly.

(3) Closure of real gaps in HsX of the CHM1 assembly and real gaps in the largest scaffold of the GM12878 assembly

The reference genomes of *S. cerevisiae* S288C and *C. elegans* Bristol were complete. However, the HsX sequence in CHM1 genome and the sequence of KQ079791 in GM12878 genome were unfinished. The HsX sequence contains 2,280 gaps with a contig N50 of 157.2 Kb. With the Pacbio reads, we performed a comparative analysis of the three tools on the closure of real gaps in the HsX assembly.

The largest sequence of KQ079791 from human GM12878 genome assembly contains 53 gaps with a contig N50 of 2.86 Mb. The performances of different tools using the Nanopore reads were compared by closing the real gaps in this scaffold.

Using LR_Gapcloser to update the reference-quality assemblies by different approaches and from large and complex genomes

In order to assess the scalability and performance of LR_Gapcloser over a range of conditions, we applied LR_Gapcloser to assemblies generated by different approaches and originating from the large and complex genomes. In the above tests, we demonstrated that more gaps were closed using the raw long reads than using the corrected reads. Therefore, in this assessment we utilized the raw reads to close the gaps.

First, we examined the performance of LR_Gapcloser to improve the contiguity of three reference-quality human genome assemblies generated from different approaches. Steinberg *et al.* utilized end-sequenced BAC clones and 100 × Illumina sequencing reads with a reference guide strategy to generate a reference-quality assembly of the CHM1 genome [23]. The assembly had a total gap length of 210.2 Mb and the contig N50 size was as low as 143.9 kb. Pendleton *et al.* generated a hybrid assembly of GM12878 cell genome with a scaffold N50 value of 26.8 Mb [29]. The contig N50 size was 1.56 Mb, and the total gap length was 146.4 Mb. Seo *et al.* generated a highly contiguous Korean genome assembly with a contig N50 size of 18.1 Mb and a scaffold N50 size of 44.8 Mb (NCBI AssemblyDB: GCA_001750385) [30]. The corresponding raw Pacbio reads for these three assemblies were downloaded from the whole genome sequencing projects and used to close gaps.

Second, we assessed the scalability of LR_Gapcloser to improve the contiguity of genome assembly of a larger size and more complex genome than the human genome. The genome of *Triticum urartu*, the progenitor of wheat A subgenome, has an estimated size of 4.94 Gb and is characterized as repeat-rich (81.4%). Ling *et al.* integrated BAC sequencing data, Illumina data, and Pacbio reads and generated the *T. Urartu* genome assembly, the size of which was 4.85 Gb. They used PBJelly and the raw Pacbio reads to finish the gaps. The final gap length was 30 Mb. The contig N50 was 278 kb (NCBI Assembly accession: GCA_003073215.1). We utilized LR_Gapcloser and the raw long reads to further close the gaps in the assembly.

Performance comparison of different hybrid assembly strategies by using TGS and NGS reads

In the genome sequencing projects in which both NGS and TGS reads were generated, although many strategies integrating the assembly, scaffolding, and gap-closure were developed to generate assemblies, their performance and accuracy have not been investigated comprehensively. To provide an optimal hybrid strategy to generate a high-quality assembly, we evaluated the contiguity and correctness of six hybrid assembly strategies by combining TGS-based and NGS-based assemblers and LR_Gapcloser. Using the NGS and TGS reads in the above analysis as test data sets, six strategies were employed to generate the assemblies of *S. cerevisiae* S288C, *C. elegans* Bristol, and HsX. In Strategy 1, Platanus [48] was employed to assemble cleaned Illumina reads from paired-end and mate-pair libraries and the gaps were finished with paired-end libraries. In Strategy 2, the gaps in the assemblies generated by Strategy 1 were finally filled using LR_Gapcloser with the raw long reads. In Strategy 3, Pacbio reads were assembled using Canu [32], a scalable and accurate TGS-

based assembler. In Strategy 4, the assemblies generated by Strategy 3 were further scaffolded using mate-pair libraries, and the gaps were closed with paired-end libraries using Platanus. In Strategy 5, the gaps in the assemblies generated by Strategy 4 were finally filled using LR_Gapcloser with the raw long reads. Unlike the above step-wise hybrid assemblies, Strategy 6 used the hybridSPAdes algorithm [33] to assemble short and long reads simultaneously. We evaluated the contiguity and correctness of the assemblies generated by these six strategies compared with the reference genomes. Finally, based on the best performance strategy, we integrated the NGS and TGS reads to generate a new assembly of the human CHM1 genome.

Availability of supporting source code and requirements

Project name: LR_Gapcloser

Project home page: http://www.fishbrowser.org/software/LR_Gapcloser/

https://github.com/CAFS-bioinformatics/LR_Gapcloser

Operating system(s): linux

Programing language: perl, shell

Other requirements: none

License: GPLv3

RRID: SCR_016194

Availability of supporting data

All supporting data including gap-closed assemblies are available at [51].

Declarations

Ethics approval and consent to participate

Not applicable.

Consent for publication

Not applicable.

Competing interests

The authors declare that they have no competing interests.

Funding

This study was supported by National Natural Science Foundation of China [31672644] and the Special Scientific Research Funds for Central Non-profit Institutes, Chinese Academy of Fishery Sciences (2018HY-ZD0207 and 2018B004).

Acknowledgement

The authors would like to thank Dr. Shunichi Kosugi for providing GMcloser, and Dr. Adam C. English for providing PBJelly.

References

1. Koepfli KP, Paten B and O'Brien SJ. The Genome 10K Project: a way forward. Annual review of animal biosciences. 2015;3:57-111. doi:10.1146/annurev-animal-090414-014900.
2. Weimer BC. 100K Pathogen Genome Project. Genome announcements. 2017;5 28 doi:10.1128/genomeA.00594-17.
3. Xu P, Zhang X, Wang X, Li J, Liu G, Kuang Y, et al. Genome sequence and genetic diversity of the common carp, Cyprinus carpio. Nature genetics. 2014;46 11:1212-9. doi:10.1038/ng.3098.
4. Lien S, Koop BF, Sandve SR, Miller JR, Kent MP, Nome T, et al. The Atlantic salmon genome provides insights into rediploidization. Nature. 2016;533 7602:200-5. doi:10.1038/nature17164.
5. Tang B, Wang Q, Yang M, Xie F, Zhu Y, Zhuo Y, et al. ContigScape: a Cytoscape plugin facilitating microbial genome gap closing. BMC Genomics. 2013;14 1:289. doi:10.1186/1471-2164-14-289.
6. Chaisson MJP, Wilson RK and Eichler EE. Genetic variation and the de novo assembly of human genomes. Nature reviews Genetics. 2015;16:627. doi:10.1038/nrg3933.
7. Altemose N, Miga KH, Maggioni M and Willard HF. Genomic Characterization of Large Heterochromatic Gaps in the Human Genome Assembly. PLoS Comput Biol. 2014;10 5:e1003628. doi:10.1371/journal.pcbi.1003628.
8. Boetzer M and Pirovano W. Toward almost closed genomes with GapFiller. Genome Biol. 2012;13 6:R56. doi:10.1186/gb-2012-13-6-r56.
9. https://sourceforge.net/projects/soapdenovo2/files/GapCloser/.
10. Paulino D, Warren RL, Vandervalk BP, Raymond A, Jackman SD and Birol I. Sealer: a scalable gap-closing application for finishing draft genomes. BMC bioinformatics. 2015;16 1:230. doi:10.1186/s12859-015-0663-4.
11. de Sa PH, Miranda F, Veras A, de Melo DM, Soares S, Pinheiro K, et al. GapBlaster-A Graphical Gap Filler for Prokaryote Genomes. PloS one. 2016;11 5:e0155327. doi:10.1371/journal.pone.0155327.

12. https://github.com/bioinfomaticsCSU/GapReduce.
13. https://github.com/rikuu/Gap2Seq/.
14. Yao G, Ye L, Gao H, Minx P, Warren WC and Weinstock GM. Graph accordance of next-generation sequence assemblies. Bioinformatics. 2012;28 1:13-6. doi:10.1093/bioinformatics/btr588.
15. Kosugi S, Hirakawa H and Tabata S. GMcloser: closing gaps in assemblies accurately with a likelihood-based selection of contig or long-read alignments. Bioinformatics. 2015;31 23:3733-41. doi:10.1093/bioinformatics/btv465.
16. Eid J, Fehr A, Gray J, Luong K, Lyle J, Otto G, et al. Real-Time DNA Sequencing from Single Polymerase Molecules. Science. 2009;323 5910:133.
17. Jain M, Fiddes I, Miga KH, Olsen HE, Paten B and Akeson M. Improved data analysis for the MinION nanopore sequencer. Nature methods. 2015;12 4:351-6. doi:10.1038/nmeth.3290.
18. English AC, Richards S, Han Y, Wang M, Vee V, Qu J, et al. Mind the gap: upgrading genomes with Pacific Biosciences RS long-read sequencing technology. PLoS One. 2012;7 11:e47768. doi:10.1371/journal.pone.0047768.
19. Chaisson MJ and Tesler G. Mapping single molecule sequencing reads using basic local alignment with successive refinement (BLASR): application and theory. BMC Bioinformatics. 2012;13:238. doi:10.1186/1471-2105-13-238.
20. Kurtz S, Phillippy A, Delcher AL, Smoot M, Shumway M, Antonescu C, et al. Versatile and open software for comparing large genomes. Genome Biol. 2004;5 2:R12. doi:10.1186/gb-2004-5-2-r12.
21. Li H and Durbin R. Fast and accurate long-read alignment with Burrows-Wheeler transform. Bioinformatics. 2010;26 5:589-95. doi:10.1093/bioinformatics/btp698.
22. Li H, Handsaker B, Wysoker A, Fennell T, Ruan J, Homer N, et al. The Sequence Alignment/Map format and SAMtools. Bioinformatics. 2009;25 16:2078-9. doi:10.1093/bioinformatics/btp352.
23. Steinberg KM, Schneider VA, Graves-Lindsay TA, Fulton RS, Agarwala R, Huddleston J, et al. Single haplotype assembly of the human genome from a hydatidiform mole. Genome research. 2014;24 12:2066-76. doi:10.1101/gr.180893.114.
24. Jackman SD, Vandervalk BP, Mohamadi H, Chu J, Yeo S, Hammond SA, et al. ABySS 2.0: resource-efficient assembly of large genomes using a Bloom filter. Genome Res. 2017;27 5:768-77. doi:10.1101/gr.214346.116.
25. Schneeberger K, Ossowski S, Ott F, Klein JD, Wang X, Lanz C, et al. Reference-guided assembly of four diverse Arabidopsis thaliana genomes. Proc Natl Acad Sci U S A. 2011;108 25:10249-54. doi:10.1073/pnas.1107739108.
26. Berlin K, Koren S and Chin CS. Assembling large genomes with single-molecule sequencing and locality-sensitive hashing. 2015;33 6:623-30. doi:10.1038/nbt.3238.
27. Lam ET, Hastie A, Lin C, Ehrlich D, Das SK, Austin MD, et al. Genome mapping on nanochannel arrays for structural variation analysis and sequence assembly. Nature biotechnology. 2012;30 8:771-6. doi:10.1038/nbt.2303.
28. Xiao CL, Chen Y and Xie SQ. MECAT: fast mapping, error correction, and de novo assembly for single-molecule sequencing reads. 2017;14 11:1072-4. doi:10.1038/nmeth.4432.
29. Pendleton M, Sebra R, Pang AW, Ummat A, Franzen O and Rausch T. Assembly and diploid architecture of an individual human genome via single-molecule technologies. 2015;12 8:780-6. doi:10.1038/nmeth.3454.
30. Seo JS, Rhie A, Kim J, Lee S, Sohn MH, Kim CU, et al. De novo assembly and phasing of a Korean human genome. Nature. 2016;538 7624:243-7. doi:10.1038/nature20098.
31. Ling H-Q, Ma B, Shi X, Liu H, Dong L, Sun H, et al. Genome sequence of the progenitor of wheat A subgenome Triticum urartu. Nature. 2018;557 7705:424-8. doi:10.1038/s41586-018-0108-0.

32. Koren S, Walenz BP, Berlin K, Miller JR, Bergman NH and Phillippy AM. Canu: scalable and accurate long-read assembly via adaptive k-mer weighting and repeat separation. Genome Res. 2017;27 5:722-36. doi:10.1101/gr.215087.116.
33. Antipov D, Korobeynikov A, McLean JS and Pevzner PA. hybridSPAdes: an algorithm for hybrid assembly of short and long reads. Bioinformatics. 2016;32 7:1009-15. doi:10.1093/bioinformatics/btv688.
34. Cao H, Wu H, Luo R, Huang S, Sun Y, Tong X, et al. De novo assembly of a haplotype-resolved human genome. 2015;33 6:617-22. doi:10.1038/nbt.3200.
35. Levy S, Sutton G, Ng PC, Feuk L, Halpern AL, Walenz BP, et al. The diploid genome sequence of an individual human. PLoS biology. 2007;5 10:e254. doi:10.1371/journal.pbio.0050254.
36. Chaisson MJ, Huddleston J, Dennis MY, Sudmant PH, Malig M, Hormozdiari F, et al. Resolving the complexity of the human genome using single-molecule sequencing. Nature. 2015;517 7536:608-11. doi:10.1038/nature13907.
37. Huddleston J, Ranade S, Malig M, Antonacci F, Chaisson M, Hon L, et al. Reconstructing complex regions of genomes using long-read sequencing technology. Genome research. 2014;24 4:688-96. doi:10.1101/gr.168450.113.
38. Phillippy AM, Schatz MC and Pop M. Genome assembly forensics: finding the elusive mis-assembly. Genome Biol. 2008;9 3:R55. doi:10.1186/gb-2008-9-3-r55.
39. http://repeatmasker.org/cgi-bin/WEBRepeatMasker.
40. Clarke J, Wu HC, Jayasinghe L, Patel A, Reid S and Bayley H. Continuous base identification for single-molecule nanopore DNA sequencing. Nature nanotechnology. 2009;4 4:265-70. doi:10.1038/nnano.2009.12.
41. Mostovoy Y, Levy-Sakin M, Lam J, Lam ET, Hastie AR, Marks P, et al. A hybrid approach for de novo human genome sequence assembly and phasing. Nature methods. 2016;13 7:587-90. doi:10.1038/nmeth.3865.
42. https://www.ncbi.nlm.nih.gov/assembly.
43. Cox MP, Peterson DA and Biggs PJ. SolexaQA: At-a-glance quality assessment of Illumina second-generation sequencing data. BMC Bioinformatics. 2010;11 1:485. doi:10.1186/1471-2105-11-485.
44. https://github.com/nh13/dwgsim.
45. Li H and Durbin R. Fast and accurate short read alignment with Burrows–Wheeler transform. Bioinformatics. 2009;25 14:1754-60.
46. Jain M, Koren S, Miga KH, Quick J, Rand AC, Sasani TA, et al. Nanopore sequencing and assembly of a human genome with ultra-long reads. Nature biotechnology. 2018;36:338.
47. Gurevich A, Saveliev V, Vyahhi N and Tesler G. QUAST: quality assessment tool for genome assemblies. Bioinformatics. 2013;29 8:1072-5. doi:10.1093/bioinformatics/btt086.
48. Kajitani R, Toshimoto K, Noguchi H, Toyoda A, Ogura Y, Okuno M, et al. Efficient de novo assembly of highly heterozygous genomes from whole-genome shotgun short reads. Genome research. 2014;24 8:1384-95. doi:10.1101/gr.170720.113.
49. Chaisson MJP, Wilson RK and Eichler EE. Genetic variation and the de novo assembly of human genomes. Nature reviews Genetics. 2015;16 11:627-40. doi:10.1038/nrg3933.
50. Treangen TJ and Salzberg SL. Repetitive DNA and next-generation sequencing: computational challenges and solutions. Nature Reviews Genetics. 2011;13 1:36-46. doi:10.1038/nrg3117.
51. http://www.fishbrowser.org/software/LR_Gapcloser/.

Table 1. Gap-closure performance in the NGS-generated assemblies with Pacbio reads (for contigs ≥ 1,000 bp)

Reads	Tool	No. of contigs	Contig N50 (bp)	Contig NGA50 (bp)	Total length without gaps (bp)	Genome Fraction (%)	Misassemblies	Ratio of misassemblies to N50 (×10^{-5})	Ratio of misassemblies to NGA50 (×10^{-5})	No. of complete genes
S. cerevisiae S288C										
	Initial assembly	472	43,406	37,861	11,275,980	92.965	29	66.8	76.5	5,958
Uncorrected Pacbio reads	GMcloser	472	43,406	37,861	11,275,980	92.965	29	66.8	76.5	5,958
	PBJelly	228	129,334	97,464	12,123,886	96.044	102	78.9	104.6	6,185
	LR_Gapcloser	70	784,212	310,248	11,603,845	94.653	30	3.8	9.7	6,115
Corrected Pacbio reads	GMcloser	100	356,432	213,603	11,540,046	94.563	37	10.3	17.3	6,107
	PBJelly	436	47,692	42,356	11,313,983	93.202	31	65.0	73.2	5,977
	LR_Gapcloser	75	706,777	296,575	11,586,142	95.016	35	4.95	11.8	6,136
C. elegans Bristol										
	Initial assembly	4,256	43,175	40,534	95,538,867	95.587	73	169.1	180.1	42,116
Uncorrected Pacbio reads	GMcloser	4,256	43,175	39,576	95,538,867	95.587	157	363.6	396.7	42,116
	PBJelly	1,968	123,707	85,754	102,625,904	98.951	1480	1196.4	1725.9	43,415
	LR_Gapcloser	341	2,603,168	443,773	99,631,648	98.364	390	15.0	87.9	43,841
Corrected Pacbio reads	GMcloser	2,017	111,801	85,058	100,911,552	98.576	1,018	910.5	1196.8	43,339
	PBJelly	657	537,136	298,230	99,579,391	97.731	424	78.9	142.2	43,674
	LR_Gapcloser	402	1,570,967	498,533	99,476,660	98.159	291	18.5	58.4	43,748
H. sapiens CHM1 chromosome X										
	Initial assembly	3,433	76,525	74,285	145,883,728	97.657	116	151.6	156.2	386
Uncorrected Pacbio reads	GMcloser	3,433	76,525	74,285	145,883,728	97.657	116	151.6	156.2	386
	PBJelly	1,280	262,085	226,918	150,211,820	99.276	470	179.3	207.1	482
	LR_Gapcloser	297	2,606,228	649,184	149,690,339	98.606	470	18.0	72.4	543
Corrected Pacbio reads	GMcloser	1,427	236,652	214,405	147,443,419	98.471	248	104.8	115.7	492
	PBJelly	1,490	211,029	190,153	149,938,882	99.077	608	288.1	319.7	472
	LR_Gapcloser	556	773,990	440,314	148,084,716	98.790	486	62.8	110.4	544

Table 2. Closure on repeat-derived gaps in the reference assemblies with Pacbio reads (for contigs ≥ 1,000 bp)

Reads	Tool	No. of contigs	Contig N50 (bp)	Contig NGA50 (bp)	Total length without gaps (bp)	Genome Fraction (%)	Misassemblies	Ratio of misassemblies to N50 ($\times 10^{-5}$)	Ratio of misassemblies to NGA50 ($\times 10^{-5}$)	No. of complete genes
S. cerevisiae S288C										
	Initial assembly	299	77,840	76,116	11,598,097	95.584	0	0	0	6,190
Uncorrected Pacbio reads	GMcloser	502	79,876	120,645	20,723,339&	95.584	0	0	0	6,190
	PBJelly	67	560,335	475,256	12,629,537	99.569	51	9.1	10.7	6,390
	LR_Gapcloser	22	813,198	781,485	12,153,365	99.795	8	0.98	1	6,403
Corrected Pacbio reads	GMcloser	108	553,180	811,382	19,143,529&	99.379	13	2.35	1.6	6,388
	PBJelly	98	317,546	297,528	12,268,477	98.896	32	10.1	10.8	6,343
	LR_Gapcloser	41	666,801	555,815	12,136,999	99.684	4	0.6	0.72	6,396
C. elegans Bristol										
	Initial assembly	406	510,819	508,558	99,305,410	99.033	0	0	0	44,678
Uncorrected Pacbio reads	GMcloser	406	510,819	508,558	99,305,410	99.033	0	0	0	44,678
	PBJelly	80	2,517,082	991,194	100,899,157	98.705	308	12.2	31.1	44,393
	LR_Gapcloser	23	10,361,459	4,731,490	100,224,225	99.687	36	0.35	0.76	44,794
Corrected Pacbio reads	GMcloser	152	1,075,373	846,874	99,962,602	99.54	70	6.51	8.27	44,778
	PBJelly	125	1,585,684	892,872	100,298,205	99.619	136	8.58	15.2	44,782
	LR_Gapcloser	51	4,509,353	2,055,178	100,136,981	99.696	61	1.35	2.97	44,792
H. sapiens CHM1 chromosome X										
	Initial assembly	977	289,166	271,924	146,253,194	97.706	0	0	0	719
Uncorrected Pacbio reads	GMcloser	977	289,166	271924	146,253,194	97.706	0	0	0	719
	PBJelly	33	14,496,807	5,459,128	149,735,973	99.917	65	0.49	1.19	791
	LR_Gapcloser	12	16,029,035	8,219,635	149,673,140	99.945	50	0.31	0.61	789
Corrected Pacbio reads	GMcloser	444	718,976	715,918	148,447,825	99.090	8	1.11	1.12	754
	PBJelly	157	1,558,141	1,398,081	149,647,694	99.726	86	5.52	6.15	777
	LR_Gapcloser	39	6,778,534	4,573,022	149,621,524	99.930	45	0.66	0.98	788

&. The assembly size after gap closure is much larger than the reference genome size.

Table 3. Improvements of three reference-quality human assemblies

Accession		Gap number	No. of contigs	Total length without gaps (bp)	Total gap length (Mb)	Contig N50 (bp)	Largest contig (bp)
GCA_000306695 (CHM1 genome)	Before gap closure	40,915	40,893	2,827,653,301	210.23	143,921	7,163,879
	After gap closure	522	508	2,857,813,755	180.04	19,078,895	57,098,690
GCA_001013985 (GM12878 cell genome)	Before gap closure	2,332	21,235	3,030,222,093	146.35	1,557,716	10,883,701
	After gap closure	210	19,113	3,126,235,473	50.67	12,989,464	49,206,979
GCA_001750385 (Korean genome)	Before gap closure	264	3,096	2,866,867,749	37.34	18,080,262	76,477,139
	After gap closure	112	2,944	2,888,283,666	15.98	23,374,317	85,176,535

Table 4. Comparison of assemblies generated by different hybrid assembly strategies (for sequence ≥ 1,000 bp)

Strategy	No. of scaffolds	Scaffold N50 (bp)	No. of contigs	Contig N50 (bp)	NGA50 (bp)	Total length (bp)	Genome Fraction (%)	Mis-assemblies	Ratio of misassemblies to contig N50 (×10^{-5})	Ratio of misassemblies to to NGA50 (×10^{-5})	No. of complete genes
S. cerevisiae S288C											
Strategy1	70	782,307	472	43,406	37,861	11,275,980	92.965	29	66.8	76.6	5,958
Strategy2	70	782,811	70	782,811	361,646	11,587,408	95.015	35	4.47	9.68	6,136
Strategy3	NA	NA	26	752,311	548,251	12,242,107	99.504	22	2.92	4.01	6,381
Strategy4	20	834,245	25	751,743	548,251	12,232,978	99.498	21	2.79	3.83	6,378
Strategy5	20	834,245	22	834,245	553,541	12,241,279	99.504	27	3.24	4.88	6,382
Strategy6	81	735,436	134	286,930	188,069	11,679,475	95.82	33	11.5	17.5	6,121
C. elegans Bristol											
Strategy1	317	7,522,240	4,256	43,175	40,534	95,538,867	95.587	73	169.1	180.1	42,116
Strategy2	317	7,831,743	134	7,831,743	453,069	99,662,404	98.464	677	8.64	149.4	43,932
Strategy3	NA	NA	195	1,207,434	381,285	101,862,626	97.363	586	48.5	153.7	43,709
Strategy4	83	12,886,407	194	1,367,460	407,816	101,775,561	99.119	541	39.6	132.6	44,214
Strategy5	83	12,886,574	83	12,886,574	433,201	102,039,090	99.264	1,003	7.78	231.5	44,267
Strategy6	3,939	803,810	5,452	83,934	69,291	100,356,139	94.838	597	711.2	861.6	41,613
H. sapiens CHM1 chromosome X											
Strategy1	217	5,785,732	3433	76,525	74,285	145,883,728	97.657	116	151.6	156.2	386
Strategy2	217	5,787,692	274	2,623,679	607,964	148,184,838	98.816	563	21.5	92.6	547
Strategy3	NA	NA	344	1,486,062	718,890	151,843,469	98.466	573	38.6	79.7	573
Strategy4	141	15,922,371	338	1,486,062	718,778	151,744,013	98.451	572	38.5	79.6	573
Strategy5	141	15,922,769	156	7,868,846	970,260	152,338,003	98.750	669	8.5	68.9	592
Strategy6	Terminated, cause unclear										

Figure 1. The primary steps in LR_Gapcloser. The long reads are colored with green and purple. The long reads are fragmented into tags of the same length. The scaffolds are colored with blue and the gaps marked as skew lines. The tracks represent that the tags are aligned to the scaffolds. (A) A gap that is completely closed with a long read. (B) A gap that is filled with two reads. (C) A gap that could be partially closed by only one read.

Figure 2. MUMmerplot of new assemblies generated by Strategy 5 compared to the reference assemblies. Alignment dotplots show the structural agreements between the new assemblies (y-axis) and the reference (x-axis). Boundaries of chromosome from the reference and of scaffolds from the assemblies are represented as dotted lines (vertical and horizontal, respectively). Scaffolds are oriented and ordered to match the chromosomes using 'mummerplot' command with '--filter --layout' option. (A) Plot between the new assembly and *S. cerevisiae* reference. (B) Plot between the new assembly and *C. elegans* reference. (C) Plot between the new assembly and CHM1 HsX reference. (D) Plot between the new CHM1 assembly and GRCh38 reference.

Additional files

Additional file 1:

Figure S1: Alignment of 18 BAC sequences to the new CHM1 genome assembly.

Figure S2: The runtime of tag fragmentation and alignment

Table S1: Whole genome Illumina sequencing reads obtained from NCBI SRA data

Table S2: The statistics of used Pacbio reads

Table S3: The statistics of used Nanopore reads

Table S4. The filled bases, runtime and memory usage of closing gaps in the NGS assemblies with Pacbio reads

Table S6. Gap-closure performance in the NGS-generated assemblies with Nanopore reads (for contigs ≥ 1,000 bp)

Table S7. The filled bases, runtime and memory usage of closing gaps in the NGS assemblies with Nanopore reads

Table S9. The filled bases, runtime and memory usage of closing the repeat-derived gaps in the reference assemblies with Pacbio reads

Table S11. Closure on repeat-derived gaps in the reference assemblies with Nanopore reads (for contigs ≥ 1,000 bp)

Table S12. The filled bases, runtime and memory usage of closing the repeat-derived gaps in the reference assemblies with Nanopore reads

Table S14. The filled bases, runtime and memory usage of closing the real gaps in HsX sequence with Pacbio reads

Table S15. The filled bases, runtime and memory usage of closing the real gaps in KQ079791 with Nanopore reads

Table S17. Summary statistics of different human *de novo* assemblies

Table S18. Sequence identities of 18 BAC sequences to the new CHM1 assembly

Additional file 2:

Table S5: Quast reports on gap-closure of NGS assemblies with Pacbio reads

Additional file 3:

Table S8. Quast reports on gap-closure of NGS assemblies with Nanopore reads

Additional file 4:

Table S10. Quast reports on closures of repeat-derived gaps with Pacbio reads

Additional file 5:

Table S13. Quast reports on closures of repeat-derived gaps with Nanopore reads

Additional file 6:

Table S16. Quast reports on assemblies of three species generate by different strategies

Figure 1

Click here to download Figure Figure 1.png

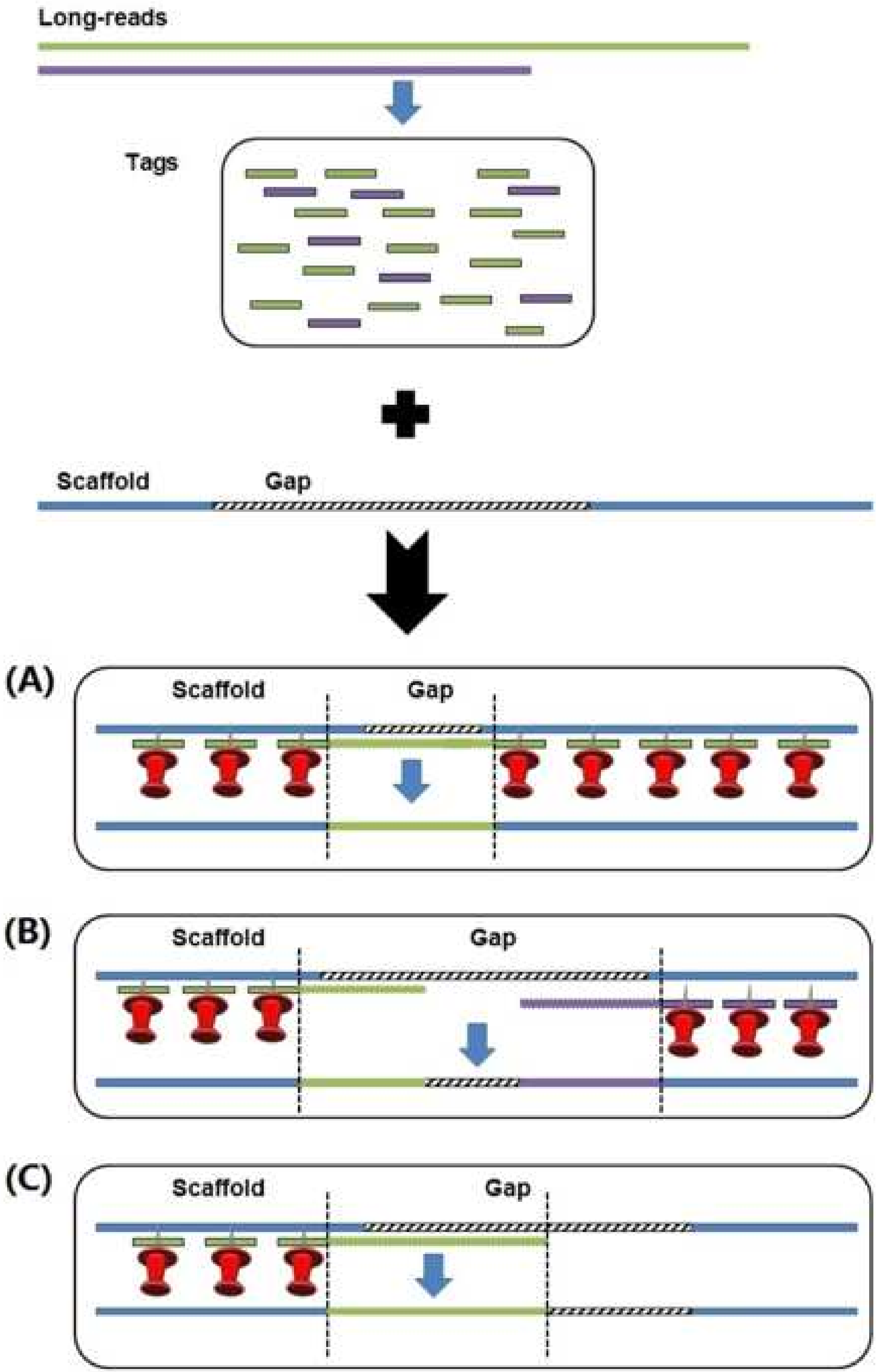

Figure 2 Click here to download Figure Figure 2.png

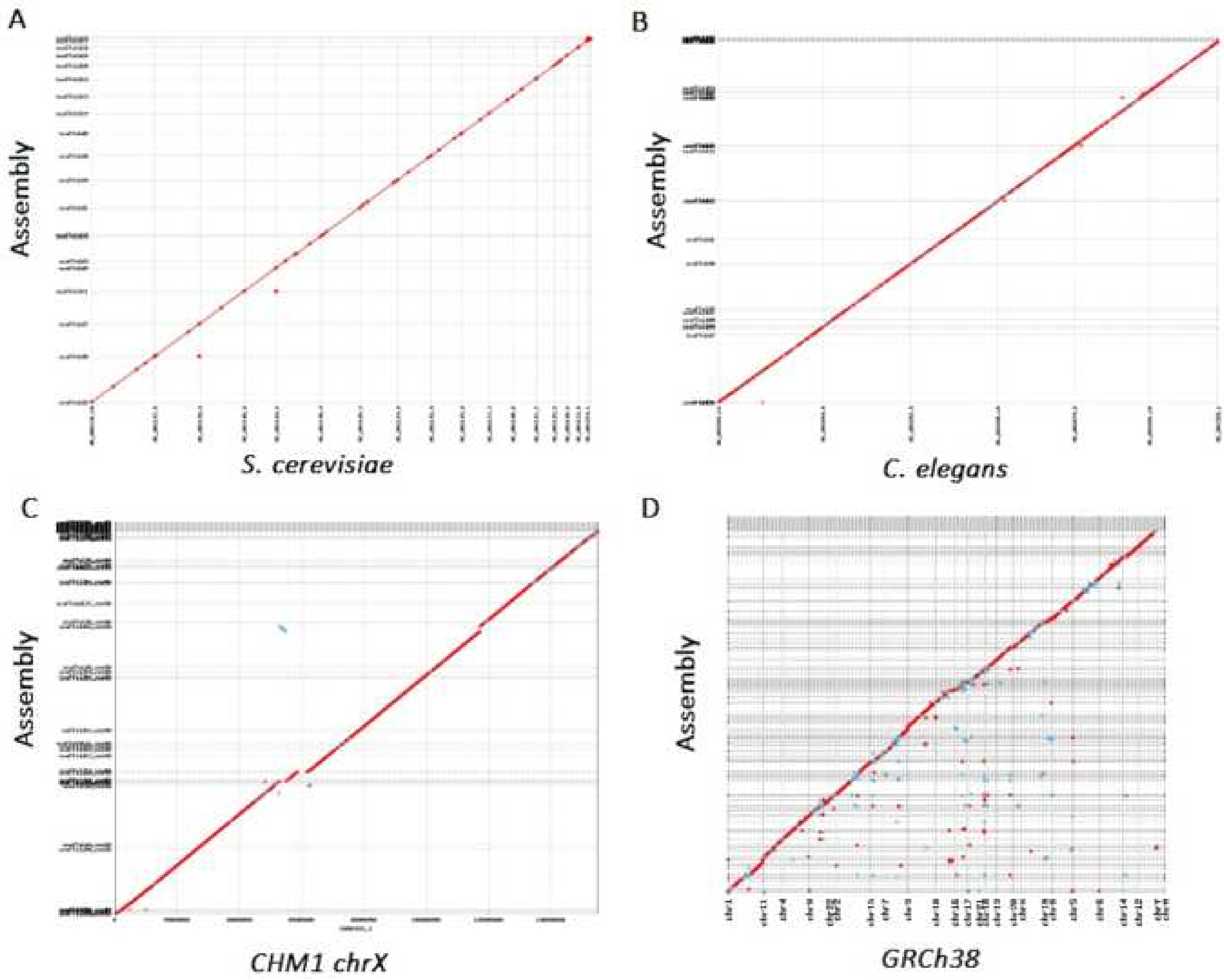

Additional file 1

Click here to access/download
Supplementary Material
Additional file 1.doc

Additional file 2

Click here to access/download
Supplementary Material
Table S5.xlsx

Additional file 3

Click here to access/download
Supplementary Material
Table S8.xlsx

Additional file 4

Click here to access/download
Supplementary Material
Table S10.xlsx

Additional file 5

Click here to access/download
Supplementary Material
Table S13.xlsx

Additional file 6

Click here to access/download
Supplementary Material
Table S16.xlsx

cover letter
Click here to download Personal Cover response letter to GIGA-D-18-00129.docx

Editorial Office of GigaScience

September 5th, 2018

Dear Dr. Nicole Nogoy,

We are grateful to three referees and you for the constructive suggestions. These suggestions greatly improve our manuscript. Thank you for invitation of the resubmission of the manuscript entitled “LR_Gapcloser: a tiling path-based gap closer that uses long reads to complete genome assembly” previously numbered GIGA-D-18-00129. We have addressed in full all the points raised by three referees and you. Major changes are highlighted in red in the revised manuscript to make them easier to find.

Sincerely,

Jiong-Tang Li, Ph.D

Key Laboratory of Aquatic Genomics, Ministry of Agriculture and Rural Affairs

Chinese Academy of Fishery Sciences

Beijing, China

Tel: +86-10-68673905, Fax: +86-10-68697522

E-mail: lijt@cafs.ac.cn

Detailed Responses to Referees' Comments from Referees and editor

Editor's Comments:

Given the reviewers' comments, there needs to be more clarity in the paper, with several aspects as the reviewers highlight in their reports (see below). We also require more comparison with other gap closing tools, and the novelty of the tool is unclear. Further, we'd also like to see how the tool performs with larger assembly datasets - larger genomes with more complexity to assess the scalability and performance of this tool - in particular, nanopore data.

Reply:

(1) We invited a professional language editing service to improve the presentation.

(2) We summarized five advantages of this tool in the Discussion section.

(3) We performed more comparisons with other tools. (i) We designed three types of gaps, including NGS-generated gaps, repeat-derived gaps, and real gaps, for comparison. (ii) In all tests, the raw Pacbio reads, corrected Pacbio reads, raw Nanopore reads, and corrected Nanopore reads were used in the comparisons. (iii) In the revised manuscript, we added the peak memory usage as an additional indictor to compare the performance of each tool.

(4) In all three types of tests listed in the manuscript, we demonstrated that our tool supports the use of Nanopore reads.

(5) We applied LR_Gapcloser into closing gaps in the assembly of the *Triticum urartu* genome (a large genome rich in repeats) and demonstrated its efficient performance on large and complex genomes.

<u>Referee 1 Comments:</u>

1. This paper describes an approach to close gaps in de novo assembled scaffolds using corrected long reads. The paper presents few novel ideas, and the English has to be improved significantly. The paper is hard to read, it has to be edited for clarity of presentation. I find that the comparisons that the paper makes to the existing software are not fair. I do not think that this paper will be of interest to wide range of researchers.

Reply:

(1) We summarized presented five advantages of this tool in the Discussion (from line 13, page 13 to line 5, page 14): (i) fast and efficient with low memory-usage; (ii) efficiently using the raw long reads (Pacbio and Nanopore) to close the gaps; (iii) producing the fewest misassemblies; (iv) efficient performance to close repeat-rich gaps; (v) suitable to close gaps in the large and complex genome assemblies.

(2) We invited a professional language editing service for clarity of presentation.

(3) In the Result section of the revised manuscript, we performed the fair comparisons to demonstrate the good performance of our tool. Either uncorrected reads or corrected reads were directly fed into these three tools (from line 27, page 15 to line 19, page 16).

(4) This fast and memory-efficient tool, LR_Gapcloser, significantly improved the contig N50 size. For instance, the contig N50 size of human CHM1 reference assembly was improved from 143 kb to 19 Mb, with a 132-fold increase. Considering that Nanopore and Pacbio reads have been widely applied into genome assembly and that LR_Gapcloser exhibits five notable advantages, we believe that this tool will be of interest to wide range of researchers.

2. The clarity of the presentation is needs to be improved significantly. The reads are split in "tags", but the paper never mentions the tag lengths, and coverage. Are the tags overlapping? In the coverage formula, what is the "insert base length", it is never explained in the text. When tag orientation is examined, how much more tags are needed to be in one direction compared to the other? What it we have 20 tags in one direction and 19 in the other -- this surely indicates a problem, but the paper never mentions on how to deal with it.

Reply:

(1) We described the tag length (line 9, page 4) and coverage (line 19, page 4).

(2) These tags are not overlapping (line 10, page 4).

(3) We explained the "insert base length" (from line 16 to line 19, page 4).

(4) We clearly described how to examine the tag orientation (from line 23, page 4 to line 1, page 5). If one alignment orientation had more uniquely aligned tags than the other orientation, then all alignments of the former orientation were retained and alignments of the latter orientation were removed. That is, we only used the partial sequence of one read to close gap. If there were 20 tags in one direction and 19 in the other, these 20 tags were retained and the other 19 tags were removed.

(5) The strategy of refining the orientations of uniquely aligned tags is one step of LR_Gapcloser. Other steps within our tool could further ensure the accuracy of our tool. All the comparisons demonstrated the high accuracy of LR_Gapcloser, suggesting the reliability of this refinement strategy.

3. The method described in the paper relies on using corrected long reads, and it does not involve computing consensus over gaps. This is a big difference that impacts run time of this method compared to PBJelly tool which is designed to be used with uncorrected long reads which are harder to map, and

also require computing consensus over gaps. For fairness of timing comparisons, time spent in correcting the reads should be added to the timings of LR_Gapcloser. Also the quality of the gap consensus is not due to LR_Gapcloser but rather reflects the performance of MECAT consensus compared to PBJelly consensus.

Reply:

(1) Our tool supports the use of the uncorrected reads to close gaps (Table 1, 2, S6, S11, S14, and S15).

(2) To perform pair comparisons, we directly fed the raw long reads (Pacbio and Nanopore reads) to each tool (from line 16 to line 19, page 17).

(3) Using the raw long reads to close gaps, all comparisons in this study demonstrated that LR_Gapcloser was much faster than PBJelly and GMcloser (Table S4, S7, S9, S12, S14, and S15).

(4) Using either the raw long reads or error-corrected long reads, LR_Gapcloser produces fewer misassemblies than PBJelly (Table 1 and 2). These results demonstrated that the high quality of LR_Gapcloser is due to its algorithm, independent of the long read correction process.

4. The paper did not convince me that there is any advantage in using LR_Gapcloser compared to a much simpler approach of mapping the corrected reads to an assembly with a fast multi-threaded tool such as MUMmer4 (https://mummer4.github.io/), picking the best alignment for every corrected read using "delta-filter -q" which is part of the mummer4 package and writing a simple script to pick the best gap close or extension into the gap of mapped reads.

Reply:

(1) The strategy of GMcloser (https://sourceforge.net/projects/gmcloser/) resembles the approach suggested by the reviewer. GMcloser splits scaffolds into sub-contigs, and aligns long reads to sub-contigs using MUMmer [1]. It selects the best alignment using "deleta-filter" in the MUMmer package. It uses likelihood-based classifiers to correctly assign long reads to gap regions of scaffolds (for details, please see the manual in 'https://sourceforge.net/projects/gmcloser/files/').

(2) All comparisons revealed that the strategy used by GMcloser closes fewer gaps and requires longer runtime and higher memory usage than LR_Gapcloser (Table 1, 2, S4, S7, S9, S12, S14, and S15).

Referee 2 Comments:

The authors of the paper "LR_Gapcloser: a tilling path-based gap closer with long reads towards complete genomes" describes a fast and very promising gap closer based on PacBio reads. This targets the very last step of the assembly process, which is to attempt to fill the gaps introduced during scaffolding. In the following I list my questions and concerns:

1. I noticed that you included simulated data in the benchmark data sets. I wonder why and what happens if that is not included.

Reply:

(1) We selected the reads and reference genomes by implementing the strict criteria: (i) the genome is complete or almost finished to facilitate the measurement of the gap-closure accuracy. (ii) Pacbio long reads (or Nanopore reads), Illumina reads, and the reference genome assembly should be generated from the same strain (from line 7 to line 14, page 15). We finally selected *S. cerevisiae* S288C, *C. elegans* Bristol, and *H. sapiens* CHM1. Although paired-end Illumina reads from these three species were available, there were lack of mate-pair reads (Table S1), leading to short scaffolds.

(2) To supplement the mate-pair reads, we adopted Boetzer *et al*'s strategy [2], which included simulated mate-pair reads, to assess the gap-closure performance of GapFiller.

(3) Following the reviewer's suggestion, we *de novo* assembled human CHM1 chromosome X with the real pair-end reads. The scaffold N50 size and contig N50 size were 137 kb and 12.4 kb, respectively. Then we ran three tools with 30 threads on the same machine to close the gaps. As shown in the following table, using either the raw Pacbio reads or corrected reads, LR_Gapcloser has the best performance and requires shortest runtime and lowest memory usage.

Table. The filled bases, runtime and memory usage of closing gaps in human CHM1 chromosome X with Pacbio reads

Reads	Tool	Filled bases (bp)	Remaining gap length (bp)	Runtime	Peak memory
	Initial assembly	0	5,758,954		
Uncorrected Pacbio reads	GMcloser	0	5,776,065	22 hours 12 minutes	165 Gb
	PBJelly	5,570,116	188,838	96 hours 55 minutes	84 Gb
	LR_Gapcloser	5,738,543	20,411	4 hours 32 minutes	10 Gb
Corrected Pacbio reads	GMcloser	4,500,457	1,258,497	32 hours	66 Gb
	PBJelly	5,225,669	533,285	17 hours 42 minutes	24 Gb
	LR_Gapcloser	5,447,891	311,063	1 hour 50 minutes	4.8 Gb

2. Your algorithm seems very greedy and I hope you can comment how sensitive it is to inaccurate gap prediction sizes. This is especially true for larger gaps where you just append reads on each end. Usually the de novo assembly often has a good reason to not proceed in this direction.

Reply: We described the comparison between the length of the added sequence and the predicted gap size (from line 18 to line 24, page 5). If the length derivation between them is under an allowed threshold, we considered that this predicted gap size was accurate and the added sequence could be filled into this gap.

3. I would like to know more about your definition of misassembly. Usually this concept is used to show that e.g. two scaffolds are wrongly joined, which does not make sense here.

Reply: We employed Quast [3] to evaluate the quality of gap-closure. As described from line 3 to line 7, page 17, Quast defines one misassembly in one contig as a position where: (i) the left flanking sequence and the right flanking sequence are over 1 kb apart on the same reference sequence, or (ii) these two flanking sequences have an overlapping region over 1 kb, or (iii) they align on opposite strands of the same genomic sequence or two different chromosomes. Please see Quast manuscript for details.

4. I would be interested why you choose 50 threads. Certainly Blasr and other methods do not fully leverage this so I would like to see how your program and others perform on more realistic e.g. 5 threads.

Reply:

(1) Read alignment and gap-closure process are much time-consuming [4, 5]. Hence, the reality is that as many threads as possible are required to close gaps.

(2) Both PBJelly and GMcloser support multiple threads. During our comparison, we ran each tool with 30 threads on the same machine to decrease the runtime.

(3) With 30 threads, PBJelly and GMcloser require even many days to close gaps (Table S4, S7, S9, S12, S14, and S15). If PBJelly and GMcloser ran with only 5 threads, they must use more days.

5. I would encourage you to be also more precise. In the result section you often talk about thresholds or "close" but don't give numbers. These would help the reader to follow and understand.

Reply: In the revised manuscript, we provided the detailed number of each threshold.

6. Can you comment on the identity of the gaps you are closing? Especially for GCRH 38 experiment.

Reply:

(1) In the Discussion, we commented on the low misassembly of the closed gaps (from line 22 to line 25, page 13). This is one novelty of this tool.

(2) Comparing the closed gaps (NGS-generated gaps and repeat-derived gaps) in human CHM1 chromosome X sequence with the corresponding reference assembly revealed that the contigs in which gaps were closed by LR_Gapcloser had an identity over 99% (Table S5, S8, S10, and S13).

(3) We did not close gaps in GCRH38 assembly since there were no available long reads from this genome. In our study, GCRH38 assembly was employed to demonstrate a general agreement between the new CHM1 assembly and it (Figure 2D).

7. I would also encourage you to check certain phrases in the abstract and result section to improve the overall English.

Reply: We invited a professional language editing service to improve the overall English.

<u>Referee 3 Comments:</u>

In this paper, authors presented a method, LR_Gapcloser, for closing gaps in genome assemblies using long read sequences from third-generation sequencing technologies. The proposed method first cut the long read sequences into the fixed-length non-overlapping short fragments. It then aligns the short fragments to the draft assembly sequences using BWA-MEM and assigns a coverage score to each alignment. After filtering out low quality/inconsistent alignments, the proposed method employs the selected supporting reads for gap closure.

The manuscript has a number of shortcomings, ranging from Introduction and Method details to the presentation of results and conclusions. Here are several examples falling into each category.

Major comments:

1. The literature on closing gaps for genome assemblies is not well investigated in the Introduction section. There are several recent tools related to gap closing such as GapCloser utility in SOAPdenovo, GapFiller, Sealer, GapBlaster, GapReduce and Gap2Seq.

Reply: We cited these recent tools in the Introduction section (from line 9 to line 11, page 3).

2. The novelties mentioned in the manuscript are unclear. Employing long read sequencing and/or mapping technologies to improve contiguity, completeness and correctness of genome assemblies has been investigated in a number of different studies in the past few years. Further, using corrected long read sequences to fill the gaps is not considered a novelty as highlighted by the authors. The manuscript should highlight better the intellectual contribution of what is done here.

Reply:

(1) In the Discussion, we clearly summarized five novelties of this tool compared with the existing two tools (from line 13, page 13 to line 5, page 14).

(2) In the revised manuscript, we provided evidence that the tool supports the use of the raw long reads to fill the gaps (Table 1, 2, S6, S11, S14, and S15).

3. The datasets used in the experiment are in the range of small or intermediate. Authors could use larger draft assembly datasets representing genomes of larger size with more complexity to assess the scalability and performance of their method over a range of conditions.

Reply: Following the referee's suggestion, we closed the gaps in the genome assembly of *Triticum urartu*, the progenitor of wheat A subgenome (from line 4 to line 12, page 11). This genome has an estimated size of 4.94 Gb and is characterized as repeat-rich (81.4%) [6]. Although some gaps had been closed by using PBJelly, there still existed a total gap size of 30 Mb. After gap-closing using LR_Gapcloser, 11 Mb (36.7%) of novel nucleotides were added to the gap regions and the contig N50 size was increased by 40%. These data demonstrated that this method is applicable on the large-sized genomic spectrum.

4. Correcting errors in noisy long read sequences is a challenging task and requires considerable amount of time and memory. To have better comparison with other tools, the authors should consider the required time for correcting the erroneous long read sequences from the third-generation sequencing instruments. As a point of comparison, the manuscript should also benchmark how long it

takes to construct the corrected reads. There is no evaluation on the memory usage of the proposed method compared to other evaluated tools.

Reply:

(1) Our tool supports the use of uncorrected reads to close gaps. To perform pair comparisons, we directly input the raw long reads (Pacbio and Nanopore reads) to all three tools (from line 16 to line 19, page 17).

(2) All comparisons demonstrated that LR_Gapcloser was much faster than the other two tools using either the raw long reads or error-corrected reads (from line 14 to line 18, page 13; Table S4, S7, S9, S12, S14, and S15).

(3) We compared the peak memory usages of these three methods and found that LR_Gapcloser required the lowest peak memory usage (lines 18 and 19, page 13; Table S4, S7, S9, S12, S14, and S15).

5. In different hybrid assembly strategies, the author could compare their results for each strategy using other competitors of the proposed method, not only using different long-range information data and LR_Gapcloser.

Reply:

(1) In different hybrid assembly strategies, we selected Canu and Platanus to represent the TGS-based assembler and NGS-based assembler, respectively. Platanus is an efficient assembly approach using Illumina shotgun short reads and has the best assembly performance compared to other existing, state-of-the art assemblers [7]. Canu is a scalable and accurate TGS-based assembler [8].

(2) We also compared the new CHM1 genome assembly generated by Strategy 5 to other human genome assemblies using long-range physical mapping data (from line 30, page 12 to line 2, page 13). The new, highly contiguous CHM1 assembly had the largest contig N50 value (28.45 Mb) thus far (except the GRCh38 reference assembly). This value is an order of magnitude larger than the CHM1 reference assembly generated by the earlier method (143.9 kb) [9], and it is even larger than previous non-reference assemblies of the human diploid genome using long-range physical strategies [10-13] (Table S17).

Minor comments:

6. Tables in the Supplementary files are not correctly ordered. In the manuscript Table S6 refers to Runtime of three tools on gaps in the NGS assemblies while in the Additional file 1 it is numbered as Table S5.

Reply: We corrected the orders of the supplementary tables.

7. Other than BWA-MEM alignment step, other parts of gap closing pipeline could be embarrassingly parallelized that improves the runtime performance of the proposed method.

Reply: We described all parallelized processes in the gap closing pipeline (from line 6 to line 14, page 14).

8. On the GitHub page, the proposed method mistakenly named LR_Gacploser.

Reply: We corrected it.

References

1. Kurtz S, Phillippy A, Delcher AL, Smoot M, Shumway M, Antonescu C, et al. Versatile and open software for comparing large genomes. Genome Biol. 2004;5 2:R12. doi:10.1186/gb-2004-5-2-r12.
2. Boetzer M and Pirovano W. Toward almost closed genomes with GapFiller. Genome biology. 2012;13 6:R56. doi:10.1186/gb-2012-13-6-r56.
3. Gurevich A, Saveliev V, Vyahhi N and Tesler G. QUAST: quality assessment tool for genome assemblies. Bioinformatics. 2013;29 8:1072-5. doi:10.1093/bioinformatics/btt086.
4. Chaisson MJ and Tesler G. Mapping single molecule sequencing reads using basic local alignment with successive refinement (BLASR): application and theory. BMC bioinformatics. 2012;13 1:238. doi:10.1186/1471-2105-13-238.
5. Li H and Durbin R. Fast and accurate long-read alignment with Burrows–Wheeler transform. Bioinformatics. 2010;26 5:589-95. doi:10.1093/bioinformatics/btp698.
6. Ling H-Q, Ma B, Shi X, Liu H, Dong L, Sun H, et al. Genome sequence of the progenitor of wheat A subgenome Triticum urartu. Nature. 2018;557 7705:424-8. doi:10.1038/s41586-018-0108-0.
7. Kajitani R, Toshimoto K, Noguchi H, Toyoda A, Ogura Y, Okuno M, et al. Efficient de novo assembly of highly heterozygous genomes from whole-genome shotgun short reads. Genome research. 2014;24 8:1384-95. doi:10.1101/gr.170720.113.
8. Koren S, Walenz BP, Berlin K, Miller JR, Bergman NH and Phillippy AM. Canu: scalable and accurate long-read assembly via adaptive k-mer weighting and repeat separation. Genome research. 2017;27 5:722-36. doi:10.1101/gr.215087.116.
9. Steinberg KM, Schneider VA, Graves-Lindsay TA, Fulton RS, Agarwala R, Huddleston J, et al. Single haplotype assembly of the human genome from a hydatidiform mole. Genome research. 2014;24 12:2066-76. doi:10.1101/gr.180893.114.
10. Pendleton M, Sebra R, Pang AW, Ummat A, Franzen O and Rausch T. Assembly and diploid architecture of an individual human genome via single-molecule technologies. 2015;12 8:780-6. doi:10.1038/nmeth.3454.
11. Seo JS, Rhie A, Kim J, Lee S, Sohn MH, Kim CU, et al. De novo assembly and phasing of a Korean human genome. Nature. 2016;538 7624:243-7. doi:10.1038/nature20098.
12. Cao H, Wu H, Luo R, Huang S, Sun Y, Tong X, et al. De novo assembly of a haplotype-resolved human genome. 2015;33 6:617-22. doi:10.1038/nbt.3200.
13. Levy S, Sutton G, Ng PC, Feuk L, Halpern AL, Walenz BP, et al. The diploid genome sequence of an individual human. PLoS biology. 2007;5 10:e254. doi:10.1371/journal.pbio.0050254.
