# Supplementary material for: LR_Gapcloser: a tiling path-based gap closer that uses long reads to complete genome assembly
Source: Gigascience. 2018 Dec 21;8(1):giy157. doi: 10.1093/gigascience/giy157 (PMC6324547; doi:10.1093/gigascience/giy157)
Supplement: response_to_reviewer_comments_original_submission.pdf [file giy157_response_to_reviewer_comments_original_submission.pdf]

Author's Response To Reviewer Comments

Close

Detailed Responses to Comments from Referee and editor
Editor’s Comments:
Please register any new software application in the SciCrunch.org database to receive a RRID (Research Resource Identification Initiative ID) number, and include this in your manuscript. This will facilitate tracking, reproducibility and re-use of your tool.
Reply: The RRID of LR_Gapcloser was listed in the section of “Availability of supporting source code and requirements”.

Referee 1 Comments:
1.For the misassemblies: Quast also reports the type of misassembly: relocations, translocations and inversions. It would be great to be able to distinguish these as these indicate different problems. E.g. an inversion can be caused by joining a short contig to an existing sequence which might be repetitive and thus hard to place correctly, while a translocation/relocations is more likely to join two completely different regions together. The latter is a more critical problem.
Reply:
(1) We reported the numbers of these three misassembly types in each assembly (line 10, page 17; Supplementary Table S5, S8, S10, S13, and S16).
(2) Quast defines one misassembly in one contig as a position where: (i) the left flanking sequence and the right flanking sequence are over 1 kb apart on the same reference sequence, or (ii) these two flanking sequences have an overlapping region over 1 kb, or (iii) they align on opposite strands of the same genomic sequence, or (iv) two different chromosomes. The misassemblies were further classified by Quast into three groups, including relocation (i and ii), inversions (iii), and translocations (iv) (Please see the manual in ‘http://quast.bioinf.spbau.ru/manual.html#sec3.1.2’). The inversion/translocation had either orientation error or chromosome error. Thus, they were more critical than the relocation event.
(3) We found that the relocation event was the major misassembly type among all three tools (Supplementary Table S5, S8, S10, S13, and S16).

2.I am sometimes confused by the usage of tags vs. alignments in the description of the method. Maybe this can be improved.
Reply: We clear described that a tag was a short sequence in one long read (line 9, page 4), different from the alignment.

3.I think, but I did not found it to be explicit, you are often using contig assemblies from short reads as a start. Is that true? I don't think so but be more explicit what the starting assembly was constructed of. If so why don't you use the scaffolds. In the same matter, have you tried to run your gab closer on a pure pacbio assembly?
Reply:
(1)We clear stated that we closed the gaps in the scaffolds in the Methods (line 27, page 17).
(2) The pure pacbio assembly generated by Strategy 3 (line 5, page 20) consists of contigs without gaps (as shown in Table 4). Thus, LR_Gapcloser could not be run on it.

4. I don't like the number or percentage of nucleotides that were filled. I would encourage you to also report the # of gabs. For example, worst case you have one very long gap and several tiny once and you only close the long gab you can still report a high success rate with the % of nucleotides closed.
Reply:
(1) The closed gap number is one indicator to estimate the performance of each tool (line 26, page 16).
(2) We reported the gap number in each assembly (Supplementary Table S5, S8, S10, and S13). This indicator of 'closed gap number' supports the best performance of LR_Gapcloser.

5. I frankly don't understand the link between LR_Gapcloser and the section about hybrid genome assembly strategies. Therefore, this section seems very independent and misplaced.
Reply: The gap-closure process is the last but most essential step in the assembly process to increase the completeness and contiguity of genome assemblies. Since that LR_Gapcloser has the best gap-closure performance, for the genomes that were sequenced using both NGS and TGS technologies, we provided an optimal hybrid assembly strategy including LR_Gapcloser.

6.In the discussion you are saying: "In the best case, an entire chromosome was assembled into a single sequence from telomere to centromere." I would first ask if that was assembled through the centromere to reach the other telomere again. Only then it would be the entire chromosome, which based on this sentence is not the case. Furthermore, please say if your sequence is a contig or scaffold as this makes a big difference.
Reply:
(1)The best case produced by the optimal hybrid strategy is that the yeast genome assembly consists of 20 contigs and covers 99.5% of the reference assembly (Table 4, Supplementary Table S16), suggesting that few regions have not been covered. The previous statement was modified as "In the best case, a reference chromosome was almost completely covered by one assembled contig.".
(2) In this best case, all gaps in the scaffolds of yeast assembly were finished. Therefore, the sequence is a contig.

7.Either I missed it or its not included: A paragraph about what coverage is needed would be great.
Reply: Previously we clearly described the coverage threshold (line 20, page 4).

Close
