# Supplementary material for: LR_Gapcloser: a tiling path-based gap closer that uses long reads to complete genome assembly
Source: Gigascience. 2018 Dec 21;8(1):giy157. doi: 10.1093/gigascience/giy157 (PMC6324547; doi:10.1093/gigascience/giy157)
Supplement: reviewer_1_report_(original_submission) -- Fritz Sedlazeck — 9/25/2018 Reviewed [file giy157_reviewer_1_report_(original_submission).pdf]

Reviewer Report

Title: LR_Gapcloser: a tiling path-based gap closer that uses long reads to complete genome assembly

Version: Original Submission **Date:** 9/25/2018

Reviewer name: Fritz Sedlazeck

Reviewer Comments to Author:

The authors of the manuscript "LR_Gapcloser: a tiling path-based gap closer that uses long reads to complete genome assembly" describe a new and fast way to improve de novo assemblies based on long reads. While there are a couple of methods existing they are often very slow so it is important to fully leverage the more expensive long reads and to have a turn around time of several hours instead of days. Overall the manuscript is well written and easy to follow. The responses to my questions in the previous review were satisfying. In the following, I list my remaining questions and concerns:

1. For the misassemblies: Quast also reports the type of misassembly: relocations, translocations and inversions. It would be great to be able to distinguish these as these indicate different problems. E.g. an inversion can be caused by joining a short contig to an existing sequence which might be repetitive and thus hard to place correctly, while a translocation/relocations is more likely to join two completely different regions together. The latter is a more critical problem.
2. I am sometimes confused by the usage of tags vs. alignments in the description of the method. Maybe this can be improved
3. I think, but I did not found it to be explicit, you are often using contig assemblies from short reads as a start. Is that true? I don't think so but be more explicit what the starting assembly was constructed of. If so why don't you use the scaffolds. In the same matter, have you tried to run your gab closer on a pure pacbio assembly?
4. I don't like the number or percentage of nucleotides that were filled. I would encourage you to also report the # of gabs. For example, worst case you have one very long gab and several tiny once and you only close the long gab you can still report a high success rate with the % of nucleotides closed.
5. I frankly don't understand the link between LR_Gapcloser and the section about hybrid genome assembly strategies. Therefore, this section seems very independent and misplaced.
6. In the discussion you are saying: "In the best case, an entire chromosome was assembled into a single sequence from telomere to centromere. " I would first ask if that was assembled through the centromere to reach the other telomere again. Only then it would be the entire chromosome, which based on this sentence is not the case. Furthermore, please say if your sequence is a contig or scaffold as this makes a big difference.
7. Either I missed it or its not included: A paragraph about what coverage is needed would be great.

Level of Interest

Please indicate how interesting you found the manuscript: Choose an item.

Quality of Written English

Please indicate the quality of language in the manuscript: Choose an item.

Declaration of Competing Interests

Please complete a declaration of competing interests, considering the following questions:

- Have you in the past five years received reimbursements, fees, funding, or salary from an organisation that may in any way gain or lose financially from the publication of this manuscript, either now or in the future?
- Do you hold any stocks or shares in an organisation that may in any way gain or lose financially from the publication of this manuscript, either now or in the future?
- Do you hold or are you currently applying for any patents relating to the content of the manuscript?
- Have you received reimbursements, fees, funding, or salary from an organization that holds or has applied for patents relating to the content of the manuscript?
- Do you have any other financial competing interests?
- Do you have any non-financial competing interests in relation to this paper?

If you can answer no to all of the above, write 'I declare that I have no competing interests' below. If your reply is yes to any, please give details below.

I declare that I have no competing interests

I agree to the open peer review policy of the journal. I understand that my name will be included on my report to the authors and, if the manuscript is accepted for publication, my named report including any attachments I upload will be posted on the website along with the authors' responses. I agree for my report to be made available under an Open Access Creative Commons CC-BY license (http://creativecommons.org/licenses/by/4.0/). I understand that any comments which I do not wish to be included in my named report can be included as confidential comments to the editors, which will not be published.

I agree to the open peer review policy of the journal
