# Supplementary material for: LR_Gapcloser: a tiling path-based gap closer that uses long reads to complete genome assembly
Source: Gigascience. 2018 Dec 21;8(1):giy157. doi: 10.1093/gigascience/giy157 (PMC6324547; doi:10.1093/gigascience/giy157)
Supplement: Supplemental Files [file giy157_supplemental_files.zip › Additional file 1.doc]

**Supplementary Figures**

**Figure S1. Alignment of 18 BAC sequences to the new CHM1 genome assembly**

(1)	(2)	(3)

(4)	(5)	(6)

(7)	(8)	(9)

(10)	(11)	(12)

(13)	(14)	(15)

(16)	(17)	(18)

Alignment dotplots using MUMmer exhibit the structural consistence between each BAC sequence (y-axis) and the new CHM1 assembly (x-axis).

**Figure S2. The runtime of tag fragmentation and alignment**

The long reads were fragmented into short tags of the same length. Under different tag length thresholds, we generated different sets of short tags. The tag sets were aligned to reference assemblies with the same parameters. The running time consisted of fragmentation time and alignment time.

**Supplementary Tables**

Table S1. Whole genome Illumina sequencing reads obtained from NCBI SRA database

| **Species (genome size^*^)** | **Read type** | **Fragment size (bp)** | **Read length (bp)** | **Genome coverage** | **Accession** |
| --- | --- | --- | --- | --- | --- |
| S. cerevisiae S288c  (12,157,105 bp) | Paired-end | 300 | 100 | 98X | ERR225691 ERR225692 |
|  | Mate-pair | 3,400 | 36 | 102X | SRR507778 |
|  | Mate-pair | 5,000 | 150 | 123X | Simulated |
|  | Mate-pair | 10,000 | 150 | 123X | Simulated |
|  | Mate-pair | 15,000 | 150 | 123X | Simulated |
| C. elegans Bristol  (100,286,401 bp) | Paired-end | 250 | 110 | 140X | DRR023912  DRR023913 |
|  | Mate-pair | 3,000 | 150 | 48X | Simulated |
|  | Mate-pair | 5,000 | 150 | 48X | Simulated |
|  | Mate-pair | 10,000 | 150 | 48X | Simulated |
|  | Mate-pair | 15,000 | 150 | 48X | Simulated |
| H. sapiens CHM1 chrX  (HsX, 155,181,468 bp) | Paired-end | 300 | 100 | 65X | SRR642643 SRR642637  SRR642642 SRR642640  SRR642641 SRR642639 |
|  | Mate-pair | 3,000 | 100 | 18X | SRR642746 SRR642683  SRR642747 SRR642650  SRR642719 |
|  | Mate-pair | 8,000 | 100 | 20X | SRR642644 SRR642646  SRR642647 SRR642645  SRR642751 |
|  | Mate-pair | 10,000 | 150 | 39X | Simulated |
|  | Mate-pair | 15,000 | 150 | 39X | Simulated |

* Genome size: a total length of the reference sequence.

**Table S2. The statistics of used Pacbio reads**

|  | **Species** | **Average length (bp)** | **N50 length (bp)** | **Genome coverage** | **Shortest length (bp)** | **Longest**  **length (bp)** | **Accession** |
| --- | --- | --- | --- | --- | --- | --- | --- |
| Uncorrected reads | S. cerevisiae | 5,727 | 18,066 | 234 X | 1 | 94,868 | ERR1655119, [ERR1655125](https://trace.ncbi.nlm.nih.gov/Traces/sra/?run=ERR1655125), [ERR1655118](https://trace.ncbi.nlm.nih.gov/Traces/sra/?run=ERR1655118) |
|  | C. elegans | 10,958 | 16,572 | 74 X | 35 | 55,460 | <http://datasets.pacb.com.s3.amazonaws.com/2014/c_elegans/list.html> |
|  | H. sapiens CHM1 assembly | 7,447 | 10,731 | 53 X | 35 | 42,774 | http://www.cbcb.umd.edu/software/PBcR/MHAP/ |
|  | chrX from CHM1 assembly | 7,941 | 11,025 | 58 X | 40 | 39,256 |  |
| Corrected reads | S. cerevisiae | 9,327 | 9,920 | 32 X | 5,000 | 25,753 |  |
|  | C. elegans | 13,127 | 17,549 | 26 X | 500 | 42,199 |  |
|  | chrX from CHM1 assembly | 5,172 | 10,410 | 18.6 X | 500 | 36,746 |  |

**Table S3. The statistics of used** Nanopore reads

|  | **Species** | **Average length (bp)** | **N50 length (bp)** | **Genome coverage** | **Shortest length (bp)** | **Longest length (bp)** | **Accession** |
| --- | --- | --- | --- | --- | --- | --- | --- |
| Uncorrected reads | S. cerevisiae | 8,000 | 11,276 | 37 X | 104 | 217,290 | ERR1883389, ERR1883402, ERR1883399, ERR1883400, ERR1883401 |
|  | C. elegans | 11,537 | 19,868 | 90 X | 90 | 134,099 | ERR2092776, ERR2092777 |
|  | H. sapiens GM12878 assembly | 6,432 | 10,589 | 29 X | 5 | 514,461 | https://github.com/nanopore-wgs-consortium/NA12878/blob/master/Genome.md |
|  | KQ079791 from GM12878 assembly | 9,774 | 13,144 | 74 X | 2,000 | 514,461 |  |
| Corrected reads | S. cerevisiae | 9,781 | 10,300 | 18 X | 5,000 | 41,191 |  |
|  | C. elegans | 28,088 | 27,529 | 44 X | 1,009 | 115,756 |  |
|  | KQ079791 from GM12878 assembly | 8,038 | 10,849 | 23 X | 1,001 | 253,568 |  |

**Table S4. The filled bases, runtime and memory usage of closing gaps in the** NGS assemblies with Pacbio reads

| **Reads** | **Tool** | **Longest gap**  **length (bp)** | **filled bases (bp)^#^** | **Remaining gap length (bp)** | **Runtime** | **Peak Memory** |
| --- | --- | --- | --- | --- | --- | --- |
| *S. cerevisiae* | | | | | | |
|  | Initial assembly | 13,533 | 0 | 290,878 |  |  |
| Uncorrected Pacbio reads | GMcloser | 13,533 | 0 | 290,878 | 24 hours 30 minutes | 69 Gb |
|  | PBJelly | 5,988 | 279,599 | 11,279 | 52 hours 6 minutes | 14 Gb |
|  | LR_Gapcloser | 74 | 290,804 | 74 | 24 minutes | 2.6 Gb |
| Corrected  Pacbio reads | GMcloser | 13,314 | 208,879 | 81,999 | 1 hour 17 minutes | 10 Gb |
|  | PBJelly | 11,336 | 226,889 | 63,989 | 1 hour 43 minutes | 9.5 Gb |
|  | LR_Gapcloser | 118 | 290,393 | 485 | 7 minutes | 799 Mb |
| *C. elegans* | | | | | | |
|  | Initial assembly | 12,580 | 0 | 3,673,023 |  |  |
| Uncorrected Pacbio reads | GMcloser | 12,580 | 105 | 3,672,918 | 59 hours 11 minutes | 199 Gb |
|  | PBJelly | 10,063 | 3,605,029 | 67,994 | 119 hours 40 minutes | 54 Gb |
|  | LR_Gapcloser | 852 | 3,661,464 | 11,559 | 4 hours 25 minutes | 6.4 Gb |
| Corrected Pacbio reads | GMcloser | 12,055 | 3,120,793 | 552,230 | 13 hours 8 minutes | 79 Gb |
|  | PBJelly | 11,898 | 3,300,285 | 372,738 | 40 hours 16 minutes | 17 Gb |
|  | LR_Gapcloser | 5,735 | 3,626,794 | 46,229 | 52 minutes | 5.3 Gb |
| HsX (CHM1) | | | | | | |
|  | Initial assembly | 13,614 | 0 | 2,052,267 |  |  |
| Uncorrected Pacbio reads | GMcloser | 13,614 | 0 | 2,053,519* | 17 hours 4 minutes | 245 Gb |
|  | PBJelly | 10,084 | 1,947,934 | 104,333 | 11 hours 55 minutes | 60 Gb |
|  | LR_Gapcloser | 1,344 | 2,028,622 | 23,645 | 3 hours 53 minutes | 11.8 Gb |
| Corrected Pacbio reads | GMcloser | 10,311 | 1,107,451 | 944,816 | 36 hours 44 minutes | 63 Gb |
|  | PBJelly | 11,642 | 1,662,159 | 390,108 | 6 hours 51 minutes | 22 Gb |
|  | LR_Gapcloser | 2,270 | 1,938,646 | 113,621 | 1 hour 30 minutes | 5.2 Gb |

#: The filled base number was the difference between the initial gap length and the remaining ‘N’ number.

* GMcloser possibly induced new gaps into the assembly.

**Table S5. Quast reports on gap-closure of NGS assemblies with Pacbio reads**

**Table S6. Gap-closure performance in the NGS-generated assemblies with Nanopore reads (for contigs ≥ 1,000 bp)**

| **Reads** | **Tool** | **No. of contigs** | **Contig N50 (bp)** | **Contig NGA50**  **(bp)** | **Total length without gaps**  **(bp)** | **Genome**  **Fraction**  **(%)** | **Misassemblies** | **Ratio of**  **misassemblies**  **to N50 (×10^-5^)** | **Ratio of**  **misassemblies**  **to NGA50 (×10^-5^)** | **No. of complete genes** |
| --- | --- | --- | --- | --- | --- | --- | --- | --- | --- | --- |
| *S. cerevisiae* | | | | | | | | | | |
|  | Initial assembly | 472 | 43,406 | 37,861 | 11,275,980 | 92.965 | 29 | 66.8 | 76.5 | 5,958 |
| Uncorrected Nanopore reads | GMcloser | 472 | 43,406 | 37,861 | 11,275,980 | 92.965 | 29 | 66.8 | 76.5 | 5,958 |
|  | PBJelly | 208 | 133,388 | 108,296 | 11,880,046 | 96.156 | 42 | 31.5 | 38.8 | 6,190 |
|  | LR_Gapcloser | 70 | 782,230 | 314,254 | 11,579,773 | 94.464 | 33 | 4.22 | 10.5 | 6,081 |
| Corrected Nanopore reads | GMcloser | 463 | 43,406 | 37,861 | 11,294,840 | 93.064 | 29 | 66.8 | 76.5 | 5,968 |
|  | PBJelly | 198 | 123,964 | 97,996 | 11,650,244 | 95.21 | 42 | 33.9 | 42.9 | 6,133 |
|  | LR_Gapcloser | 75 | 744,960 | 284,592 | 11,571,811 | 94.955 | 43 | 5.77 | 15.1 | 6,125 |
| *C. elegans* | | | | | | | | | | |
|  | Initial assembly | 4,256 | 43,175 | 40,534 | 95,538,867 | 95.587 | 73 | 169.1 | 180.1 | 42,116 |
| Uncorrected Nanopore reads | GMcloser | 4,260 | 43,175 | 39,576 | 95,545,583 | 95.595 | 157 | 363.6 | 396.7 | 42,117 |
|  | PBJelly | 1,961 | 125,916 | 91,823 | 102,881,399 | 98.764 | 786 | 624.2 | 856.0 | 43,423 |
|  | LR_Gapcloser | 339 | 2,930,410 | 537,820 | 99,475,078 | 97.798 | 435 | 14.8 | 80.9 | 43,608 |
| Corrected Nanopore reads | GMcloser | 3,288 | 50,684 | 46,851 | 97,220,441 | 96.747 | 294 | 580.1 | 627.5 | 42,698 |
|  | PBJelly | 4,255 | 43,175 | 39,586 | 95,541,606 | 95.59 | 157 | 363.6 | 396.6 | 42,118 |
|  | LR_Gapcloser | 460 | 963,758 | 390,559 | 99,367,228 | 98.707 | 468 | 48.6 | 119.8 | 43,989 |

**Table S7. The filled bases, runtime and memory usage of closing gaps in the NGS assemblies with Nanopore** reads

| **Reads** | **Tool** | **Longest gap**  **length (bp)** | **filled bases (bp)** | **Remaining gap length (bp)** | **Runtime** | **Peak Memory** |
| --- | --- | --- | --- | --- | --- | --- |
| *S. cerevisiae* | | | | | | |
|  | Initial assembly | 13,533 | 0 | 290,878 |  |  |
| Uncorrected Nanopore reads | GMcloser | 13,533 | 0 | 290,878 | 58 minutes | 11 Gb |
|  | PBJelly | 5,777 | 272,593 | 18,285 | 4 hours 29 minutes | 4.1 Gb |
|  | LR_Gapcloser | 90 | 290,788 | 90 | 7 minutes | 780 Mb |
| Corrected Nanopore reads | GMcloser | 13,533 | 12,235 | 278,643 | 25 minutes | 6.1 Gb |
|  | PBJelly | 13,533 | 217,381 | 73,497 | 1 hour 25 minutes | 1.5 Gb |
|  | LR_Gapcloser | 215 | 290,159 | 719 | 4 minutes | 450 Mb |
| *C. elegans* | | | | | | |
|  | Initial assembly | 12,580 | 0 | 3,673,023 |  |  |
| Uncorrected Nanopore reads | GMcloser | 12,580 | 6,043 | 3,666,980 | 162 hours | 241 Gb |
|  | PBJelly | 6,882 | 3,625,049 | 47,974 | 26 hours 55 minutes | 66 Gb |
|  | LR_Gapcloser | 384 | 3,661,973 | 11,050 | 5 hours 22 minutes | 14.6 Gb |
| Corrected Nanopore reads | GMcloser | 12,580 | 1,288,001 | 2,385,022 | 161 hours 40 minutes | 88 Gb |
|  | PBJelly | 12,580 | 2,469 | 3,670,554 | 11 hours 30 minutes | 27 Gb |
|  | LR_Gapcloser | 3,130 | 3,603,312 | 69,711 | 2 hours 6 minutes | 6.2 Gb |

**Table S8. Quast reports on gap-closure of NGS assemblies with Nanopore reads**

**Table S9. The filled bases, runtime and memory usage of closing the** repeat-derived gaps in the reference assemblies with Pacbio reads

| **Reads** | **Tool** | **Longest gap length (bp)** | **filled bases (bp)** | **Remaining gap length (bp)** | **Runtime** | **Peak Memory** |
| --- | --- | --- | --- | --- | --- | --- |
| *S. cerevisiae* | | | | | | |
|  | Initial assembly | 11,510 | 0 | 513,395 |  |  |
| Uncorrected Pacbio reads | GMcloser | Error (The size of the gap-closed genome, 20.7 Mb, is larger than the reference genome size, 12.1 Mb.) | | | 24 hours 27 minutes | 50 Gb |
|  | PBJelly | 6,239 | 480,814 | 32,581 | 24 hours 53 minutes | 19 Gb |
|  | LR_Gapcloser | 935 | 510,395 | 3,000 | 44 minutes | 2.5 Gb |
| Corrected  Pacbio reads | GMcloser | Error (The size of the gap-closed genome, 19.1 Mb, is larger than the reference genome size, 12.1 Mb.) | | | 19 minutes | 7.8 Gb |
|  | PBJelly | 7,587 | 368,531 | 144,864 | 57 minutes | 2.6 Gb |
|  | LR_Gapcloser | 2,533 | 495,440 | 17,955 | 10 minutes | 808 Mb |
| *C. elegans* | | | | | | |
|  | Initial assembly | 20,250 | 0 | 964,793 |  |  |
| Uncorrected Pacbio reads | GMcloser | 38,285 | 0 | 966,318 | 51 hours 30 minutes | 57 Gb |
|  | PBJelly | 30,857 | 815,442 | 149,351 | 8 hours | 54 Gb |
|  | LR_Gapcloser | 13,910 | 892,825 | 71,968 | 4 hours 8 minutes | 14.6 Gb |
| Corrected Pacbio reads | GMcloser | 23,817 | 650,515 | 314,278 | 9 hours 44 minutes | 20 Gb |
|  | PBJelly | 32,514 | 636,804 | 327,989 | 2 hours 40 minutes | 17 Gb |
|  | LR_Gapcloser | 13,910 | 824,687 | 140,106 | 1 hour 22 minutes | 5.6 Gb |
| HsX (CHM1) | | | | | | |
|  | Initial assembly | 20,638 | 0 | 3,430,913 |  |  |
| Uncorrected Pacbio reads | GMcloser | 27,157 | 0 | 3,431,548 | 23 hours 13 minutes | 155 Gb |
|  | PBJelly | 13,336 | 3,380,447 | 50,466 | 8 hours 57 minutes | 60 Gb |
|  | LR_Gapcloser | 701 | 3,418,046 | 12,867 | 5 hours 33 minutes | 11.7 Gb |
| Corrected Pacbio reads | GMcloser | 8,030 | 2,179,114 | 1,251,799 | 35 hours 53 minutes | 26 Gb |
|  | PBJelly | 14,469 | 3,119,269 | 311,644 | 2 hours 34 minutes | 22 Gb |
|  | LR_Gapcloser | 3,486 | 3,373,736 | 57,177 | 2 hours 10 minutes | 5.1 Gb |

**Table S10. Quast reports on closures of repeat-derived gaps with Pacbio reads**

**Table S11. Closure on repeat-derived gaps in the reference assemblies with Nanopore reads** (for contigs ≥ 1,000 bp)

| **Reads** | **Tool** | **No. of contigs** | **Contig N50 (bp)** | **Contig NGA50**  **(bp)** | **Total length without gaps**  **(bp)** | **Genome**  **Fraction**  **(%)** | **Misassemblies** | **Ratio of**  **misassemblies**  **to N50 (×10^-5^)** | **Ratio of**  **misassemblies**  **to NGA50 (×10^-5^)** | **No. of complete genes** |
| --- | --- | --- | --- | --- | --- | --- | --- | --- | --- | --- |
| *S. cerevisiae* | | | | | | | | | | |
|  | Initial assembly | 299 | 77,840 | 76,116 | 11,598,097 | 95.584 | 0 | 0 | 0 | 6,190 |
| Uncorrected Nanopore reads | GMcloser | 504 | 79,876 | 120,645 | 20,731,022^&^ | 95.637 | 0 | 0 | 0 | 6,191 |
|  | PBJelly | 75 | 513,875 | 513,564 | 12,231,991 | 99.197 | 30 | 5.83 | 5.84 | 6,355 |
|  | LR_Gapcloser | 34 | 778,648 | 666,050 | 12,116,338 | 99.624 | 4 | 0.51 | 0.6 | 6,386 |
| Corrected Nanopore reads | GMcloser | 488 | 79,876 | 120,645 | 20,816,492^&^ | 95.94 | 0 | 0 | 0 | 6,205 |
|  | PBJelly | 93 | 314,045 | 314,045 | 12,186,052 | 98.569 | 21 | 0.67 | 0.67 | 6,338 |
|  | LR_Gapcloser | 54 | 540,963 | 537,256 | 12,086,608 | 99.456 | 3 | 0.56 | 0.56 | 6,379 |
| *C. elegans* | | | | | | | | | | |
|  | Initial assembly | 406 | 510,819 | 508,558 | 99,305,410 | 99.033 | 0 | 0 | 0 | 44,678 |
| Uncorrected Nanopore reads | GMcloser | 406 | 510,819 | 508,558 | 99,305,410 | 99.033 | 0 | 0 | 0 | 44,678 |
|  | PBJelly | 81 | 2,627,563 | 933,591 | 100,881,171 | 99.705 | 186 | 7.1 | 19.9 | 44,786 |
|  | LR_Gapcloser | 32 | 6,330,309 | 5,652,996 | 100,215,962 | 99.481 | 26 | 0.41 | 0.46 | 44,751 |
| Corrected Nanopore reads | GMcloser | 393 | 510,819 | 508,558 | 99,362,327 | 99.061 | 10 | 1.96 | 1.97 | 44,679 |
|  | PBJelly | 103 | 1,977,420 | 892,154 | 101,276,760 | 99.775 | 322 | 16.1 | 36.1 | 44,790 |
|  | LR_Gapcloser | 68 | 2,454,206 | 1,851,410 | 100,125,766 | 99.647 | 68 | 2.77 | 3.67 | 44,773 |

&. The assembly size after gap closure is much larger than the reference genome size.

**Table S12. The filled bases, runtime and memory usage of closing the repeat-derived gaps in the reference assemblies with Nanopore reads**

| **Reads** | **Tool** | **Longest gap length (bp)** | **filled bases (bp)** | **Remaining gap length (bp)** | **Runtime** | **Peak Memory** |
| --- | --- | --- | --- | --- | --- | --- |
| *S. cerevisiae* | | | | | | |
|  | Initial assembly | 11,510 | 0 | 513,395 |  |  |
| Uncorrected Nanopore reads | GMcloser | Error (The size of the gap-closed genome, 20.7 Mb, is larger than the reference genome size, 12.1 Mb.) | | | 57 minutes | 9.1 Gb |
|  | PBJelly | 8,333 | 420,297 | 93,098 | 2 hours 37 minutes | 7.3 Gb |
|  | LR_Gapcloser | 2,327 | 491,318 | 22,077 | 12 minutes | 746 Mb |
| Corrected  Nanopore reads | GMcloser | Error (The size of the gap-closed genome, 20.8 Mb, is larger than the reference genome size, 12.1 Mb.) | | | 11 minutes | 5.1 Gb |
|  | PBJelly | 11,674 | 343,197 | 170,198 | 1 hour 19 minutes | 1.5 Gb |
|  | LR_Gapcloser | 4,214 | 458,318 | 55,077 | 7 minutes | 470 Mb |
| *C. elegans* | | | | | | |
|  | Initial assembly | 20,250 | 0 | 964,793 |  |  |
| Uncorrected Nanopore reads | GMcloser | 38,285 | 0 | 966,318 | 194 hours 51 minutes | 62 Gb |
|  | PBJelly | 32,806 | 802,790 | 162,003 | 9 hours 53 minutes | 65 Gb |
|  | LR_Gapcloser | 13,910 | 899,779 | 65,014 | 3 hours 14 minutes | 14.8 Gb |
| Corrected Nanopore reads | GMcloser | 38,285 | 32,049 | 932,744 | 127 hours 24 minutes | 31 Gb |
|  | PBJelly | 33,081 | 776,557 | 188,236 | 7 hours 12 minutes | 28 Gb |
|  | LR_Gapcloser | 13,910 | 895,082 | 69,711 | 2 hours | 6.1 Gb |

**Table S13. Quast reports on closures of repeat-derived gaps with Nanopore reads**

**Table S14. The filled bases, runtime and memory usage of closing the real gaps in HsX sequence with Pacbio reads**

| **Reads** | **Tool** | **Gap number** | **Contig N50 (bp)** | **Mean gap**  **length (bp)** | **Longest gap length (bp)** | **filled bases (bp)** | **Remaining gap length (bp)** | **Runtime** | **Peak Memory** |
| --- | --- | --- | --- | --- | --- | --- | --- | --- | --- |
|  | Initial assembly | 2,280 | 157,201 | 2,408 | 3,000,000 | 0 | 5,491,129 |  |  |
| Uncorrected Pacbio reads | GMcloser | 2,278 | 157,201 | 2,411 | 3,000,000 | 70,000 | 5,421,129 | 17 hours 37 minutes | 44 Gb |
|  | PBJelly | 1,812 | 182,664 | 2,680 | 3,000,000 | 639,935 | 4,851,194 | 5 hours 22 minutes | 60 Gb |
|  | LR_Gapcloser | 166 | 4,068,228 | 25,126 | 2,957,852 | 1,370,440 | 4,120,689 | 6 hours 10 hours | 11 Gb |
| Corrected Pacbio reads | GMcloser | 959 | 601,269 | 4,572 | 2,988,042 | 1,105,687 | 4,385,442 | 34 hours 5 minutes | 37 Gb |
|  | PBJelly | 1,896 | 179,590 | 2,638 | 3,000,000 | 487,946 | 5,003,183 | 1 hour 54 minutes | 23 Gb |
|  | LR_Gapcloser | 273 | 3,075,146 | 15,692 | 2,898,692 | 1,206,952 | 4,284,177 | 2 hours 37 minutes | 5 Gb |

**Table S15. The filled bases, runtime and memory usage of closing the real gaps in KQ079791 with Nanopore reads**

| **Reads** | **Tool** | **Gap number** | **Contig N50 (bp)** | **Mean gap**  **length (bp)** | **Longest gap length (bp)** | **filled bases (bp)** | **Remaining gap length (bp)** | **Runtime** | **Peak Memory** |
| --- | --- | --- | --- | --- | --- | --- | --- | --- | --- |
|  | Initial assembly | 53 | 2,861,236 | 52,302 | 1,808,749 | 0 | 2,772,008 |  |  |
| Uncorrected Nanopore reads | GMcloser | 53 | 2,861,236 | 52,302 | 1,808,749 | 0 | 2,772,008 | 11 hours 9 minutes | 11 Gb |
|  | PBJelly | 26 | 6,469,247 | 98,512 | 1,807,814 | 210,674 | 2,561,334 | 16 hours 9 minutes | 36 Gb |
|  | LR_Gapcloser | 19 | 6,477,330 | 114,267 | 1,793,353 | 600,925 | 2,171,083 | 2 hours 20 minutes | 4 Gb |
| Corrected Nanopore reads | GMcloser | 53 | 2,861,236 | 52,302 | 1,808,749 | 0 | 2,772,008 | 16 hours 21 minutes | 10 Gb |
|  | PBJelly | 47 | 3,070,685 | 58,205 | 1,808,749 | 36,348 | 2,735,660 | 4 hours | 13 Gb |
|  | LR_Gapcloser | 43 | 3,736,430 | 61,970 | 1,794,514 | 107,257 | 2,664,751 | 1 hour 11 minutes | 3 Gb |

**Table S16. Quast reports on assemblies of three species generate by different strategies**

**Table S17. Summary statistics of different human *de novo* assemblies**

| **Genome** | **AK1** | **CHM1**  **(new assembly)** | **CHM1** | **GM12878** | **GRCh38*** | **YH_2.0** |
| --- | --- | --- | --- | --- | --- | --- |
| Accession | GCA_001750385 |  | GCA_000306695 | GCA_001013985 | GCA_000001405 | GCA_000004845 |
| Sequencing | PacBio and BAC | PacBio and Illumina | Illumina and BAC | Pacbio | BAC and fosmid | Illumina and fosmid |
| De novo assembly approach | FALCON | Canu, Platanus, LR_Gapcloser | SRPRISM, ARGO (Reference-guided) | Celera and FALCON | Multiple methods | SOAPdenovo |
| Physical mapping | BioNano | NA | NA | BioNano | FISH, OM and fingerprint contigs | CG |
| Scaffold/contig N50 (Mb) | 44.85/18.08 | 28.45/28.45 | 50.36/0.14 | 26.83/1.56 | 59,36/56.41 | 20.52/0.02 |
| Scaffold/contig L50 | 21/46 | 31/31 | 20/5,635 | 37/532 | 17/19 | 39/40,005 |
| No. of scaffolds/contigs | 2,832/3,096 | 2,265/2,591 | 163/40,828 | 18,903/ 21,235 | 875/1,536 | 125,643/361,157 |
| No. of gaps | 264 | 326 | 40,892 | 2,332 | 349 | 235,514 |
| Total gap length (Mb) | 37.34 | 0.051 | 210.23 | 146.35 | 161,37 | 105.20 |
| Total bases/non-N bases in assembly (bp) | 2,904,207,228/  2,866,867,749 | 2,997,071,794/  2,997,020,030 | 3,037,866,619/  2,827,653,301 | 3,176,574,379/  3,030,222,093 | 3,257,347,282/  3,095,978,931 | 2,911,235,363/  2,806,031,133 |

We compared the sequencing technologies, assembly approaches, and assembly statistics of some high-quality human assemblies thus far. The statistics were available at the NCBI AssemblyDB. The comparison reveals the power of the hybrid assembly of Strategy 5 including TGS technologies and NGS technologies to produce assemblies with higher contig N50 value than non-reference assemblies generated by prior methods. * The statistic of GRCh38 includes regions with alternate loci or patches.

**Table S**18. Sequence identities of 18 BAC sequences to the new CHM1 assembly

| **Accession** | **Length (bp)** | **No. of SNPs/Indels** | **Identity (%)** |
| --- | --- | --- | --- |
| AC243499 | 199,928 | 39 | 99.98 |
| AC243585 | 193,095 | 51 | 99.97 |
| AC243586 | 221,226 | 68 | 99.97 |
| AC243621 | 243,129 | 107 | 99.96 |
| AC243629 | 232,622 | 74 | 99.97 |
| AC243650 | 239,100 | 101 | 99.96 |
| AC243654 | 227,101 | 64 | 99.97 |
| AC243658 | 223,520 | 59 | 99.97 |
| AC243659 | 217,579 | 225 | 99.90 |
| AC243685 | 200,520 | 60 | 99.97 |
| AC243734 | 209,874 | 40 | 99.98 |
| AC243742 | 216,161 | 70 | 99.97 |
| AC254824 | 223,437 | 56 | 99.97 |
| AC255380 | 195,068 | 91 | 99.95 |
| AC255406 | 214,505 | 43 | 99.98 |
| AC255440 | 230,869 | 77 | 99.97 |
| AC255484 | 208,409 | 28 | 99.99 |
| AC255547 | 217,217 | 160 | 99.93 |
